# Novel biphenyl-substituted 1,2,4-oxadiazole ferroelectric liquid crystals: synthesis and characterization

**DOI:** 10.3762/bjoc.11.26

**Published:** 2015-02-11

**Authors:** Mahabaleshwara Subrao, Dakshina Murthy Potukuchi, Girish Sharada Ramachandra, Poornima Bhagavath, Sangeetha G Bhat, Srinivasulu Maddasani

**Affiliations:** 1 Department of Chemistry, Manipal Institute of Technology, Manipal University, Manipal - 576 104, India; 2 Syngene International Ltd., Biocon Park, Bommasandra, Bangalore - 560 099, India; 3 Department of Physics, University College of Engineering, Jawaharlal Nehru Technological University: Kakinada, Kakinada - 533 003, India

**Keywords:** 3,5-disubstituted-1,2,4-oxadiazoles, ferroelectric switching, mesomorphism, optical textures, SmC* phase, spontaneous polarization, Suzuki coupling

## Abstract

Two novel series of unsymmetrically substituted 1,2,4-oxadiazole viz., R.Ox.C^*^C*_n_* compounds are synthesized and characterized. An optically active, (*S*)-(+)-methyl 3-hydroxy-2-methylpropionate is used to introduce a chiral center in the molecule. A biphenyl moiety prepared by Suzuki coupling reaction is directly attached to the oxadiazole core at C-5 position. Investigations for the phase behavior revealed that the series with a benzyl group on one end of the oxadiazole core exhibits an 1D orthogonal smectic-A phase while the second series with dodecyl flexible end chain shows orthogonal smectic-A and tilted chiral smectic-C (SmC*) phases over a wide range of temperatures. The smectic-C phase exhibits ferroelectric (FE) polarization switching. The mesomorphic thermal stabilities of these compounds are discussed in the domain of the symmetry and the flexibility of the alkyloxy end chain length attached to the chiral center.

## Introduction

The 1,2,4-oxadiazole derivatives are prevalently reported compounds with promising biological [1–5] and physiological [6–8] activity such as antiinflammatory, antibacterial, antimicrobial, antifungal, anticancer, anticonvulsant, growth hormone secretogogues, antispasmodics, antithrombotic, etc. Thus these compounds received [9] considerable attention for the last two decades in new drug invention programs. In recent years, these compounds are also exploited for various technical applications [10–15] due to their electroluminescent, non-linear optical, electron transport and liquid crystalline (LC) properties. Torgova et. al., have reported [16–19] 3,5-disubstituted-1,2,4-oxadiazoles with LC phase behavior for the first time in the literature. Subsequently, the derivatives of 1,2,4-oxadiazoles with different configurations have been reported [11,20–22] which exhibited the thermotropic liquid crystalline nematic (N), smectic and polar smectic (bent or banana) phase structures.

Due to the presence of three heteroatoms in the ring structure of the oxadiazole, it possesses a high dipole moment [23–24] and polarisability, which results for greater optical anisotropy and birefringence of the material. It is well established that the chiral center in a molecule renders it to exhibit ferroelectric (FE) behavior due to its configuration for transverse dipole moment (μ_t_). Meyer et al. [25] demonstrated the FE nature in tilted smectic-C (SmC) phase of LC materials for the first time and then proved that the FELCs are potential candidates for EO display devices owing to their large birefringence, fast response [26] and large viewing angles. The ferroelectric materials possess [27–28] spontaneous polarization due to the reduced symmetry of *C*_2_*_v_* caused by molecular polarity and thus make the phase as a polar phase. Thus, an oxadiazole derivative with a chiral center is tantamount to exhibit FE properties.

In order to explore these materials in devices, it is necessary that they have a low melting point (at ambient temperatures) and exhibit the LC phases over a wide range of temperatures. It is found in the literature [29] that the unsymmetrically substituted benzene derivatives with a biphenyl moiety possess low melting points. Hence, presently we made a methodical attempt to synthesize and characterize unsymmetrically substituted 1,2,4-oxadiazoles with a chiral substituted biphenyl moiety at C-5 position of the ring and two different moieties at C-3 position of the oxadiazole core, which are exhibiting the FELC phase structures.

## Results and Discussion

### Synthesis and characterization

**Scheme 1:**
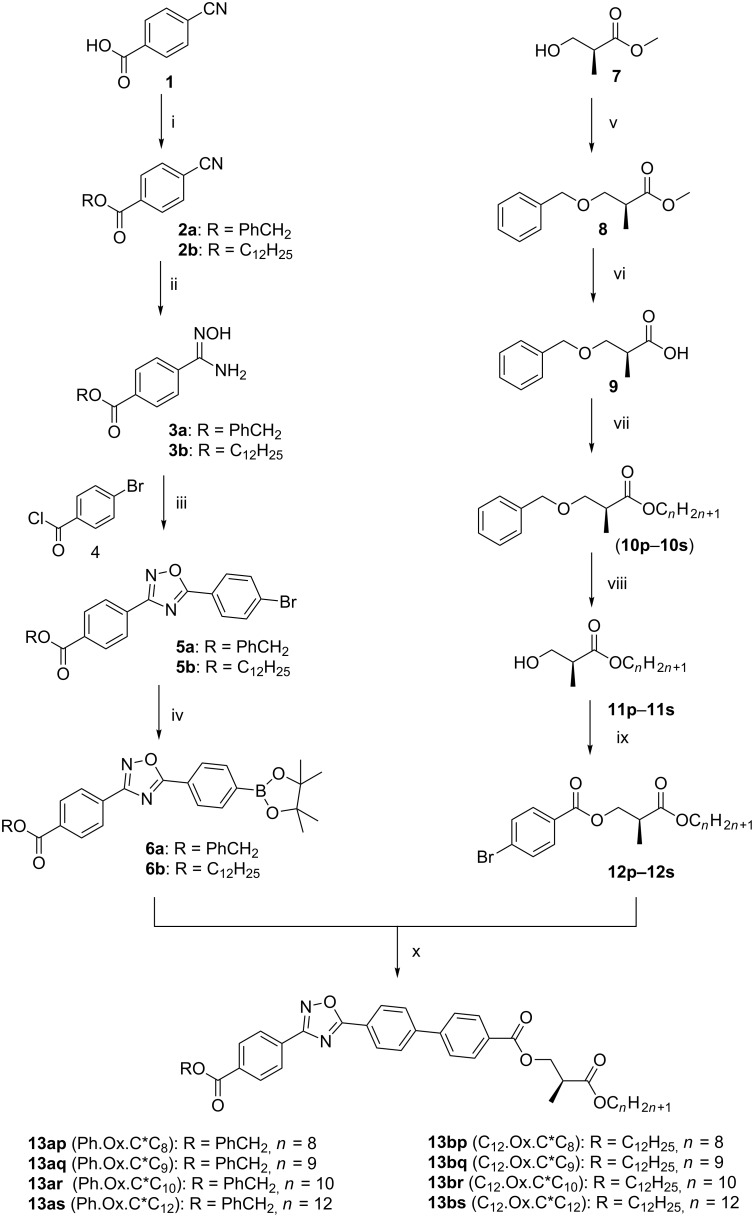
Synthetic route for the preparation of R.Ox.C*C*_n_* compounds. (i) PhCH_2_Br, K_2_CO_3_, DMF/1-dodecanol, DCC, DMAP, DCM, (ii) NH_2_OH·HCl, NaOH, EtOH, 70 °C, (iii) pyridine, 80 °C, (iv) Pin_2_B_2_, KOAc, Pd(dppf)Cl_2_, dioxane, 110 °C, (v) benzyl 2,2,2-trichloroacetimidate, CF_3_SO_3_H, *n*-hexane, rt, (vi) LiOH, MeOH, H_2_O, (vii) alkyl alcohol, DCC, DMAP, DCM, (viii) Pd/C, EtOAc, H_2_ (1 atm), (ix) 4-bromobenzoic acid, DCC, DMAP, DCM, (x) Pd(PPh_3_)_2_, Na_2_CO_3_, 1,2-DME, H_2_O, MW, 120 °C, 20 min.

The synthesis of 1,2,4-oxadiazoles involves [30] a single stage dehydration of O-acylated amidoximes via a reaction between amidoximes and derivatives of carbonyl compounds (esters, amides, acids, acid chlorides, aldehydes, etc.). Otherwise, the reaction is based on the 1,3-dipolar cycloaddition of *N*-oxides to azomethines, nitriles and iminoesters. In the present study, we synthesized 1,2,4-oxadiazoles through amidoxime intermediates with substitutions at C-3 and C-5 positions. A moiety with a chiral center is attached to the phenyl ring (at C-5 position) of oxadiazole in the molecular structure. The synthetic route for the preparation of 3,5-disubstituted-1,2,4-oxadiazoles presently adopted is presented in Scheme 1.

4-Cyanobenzoic acid is converted into its ester by treating with two different moieties, i.e., benzyl bromide and 1-dodecanol under different reaction conditions. Treatment of benzyl 4-cyanobenzoate (**2a**) with hydroxylamine hydrochloride yielded the corresponding benzyl 4-(*N*'-hydroxycarbamimidoyl)benzoate (**3a**). This amidoxime (**3a**) is converted into benzyl 4-(5-(4-bromophenyl)-1,2,4-oxadiazole-3-yl)benzoate (**5a**) by treating it with 4-bromobenzoyl chloride in the presence of pyridine. Oxadiazole **5a** is borylated under Miyaura coupling conditions [31–34] with bis(pinacolato)diborane (pin_2_b_2_) to give benzyl 4-(5-(4-(4,4,5,5-tetramethyl-1,3,2-dioxaborolan-2-yl)phenyl)-1,2,4-oxadiazol-3-yl)benzoate (**6a**), which facilitated the C–C bond formation of a biphenyl group. The same methodology is adopted to get the product **6b** which contains a dodecyl chain instead of the benzyl moiety as in **6a**. The hydroxy group of the chiral ingredient, i.e., (*S*)-(+)-methyl 3-hydroxy-2-methylpropanoate (**7**) is protected by a benzyl group by treating it with benzyl 2,2,2-trichloroacetimidate. Hydrolysis of product **8** gives the carboxylic acid **9** which on esterification with different long chain alcohols gives various esters **10p–10s**. The esters containing the benzyl ether group are deprotected to give the corresponding alcohols (**11p–11s**). Then they are treated with 4-bromobenzoic acid to give products **12p–12s** with two ester groups and a chiral center. This bromo-substituted chiral molecule is treated with boronate esters **6a/6b** under Suzuki coupling conditions to get the final products with a biphenyl moiety directly attached to the oxadiazole core at C-5 position, **13ap–13as** (Ph.Ox.C^*^C*_n_*) **/ 13bp–13bs** (C_12_.Ox.C^*^C*_n_*). The detailed procedures involved in various steps of the synthesis are explained in Supporting Information File 1. The final oxadiazole products are characterised by spectroscopic techniques. The peaks at δ_C_ = 175.29 and 168.38 ppm in the ^13^C NMR spectrum confirm the formation of the 1,2,4-oxadiazole ring without effecting [35–37] the ester group (δ_C_ = 165.76 ppm), attached to the phenyl ring under these conditions.

### Phase characterization

**Figure 1:**
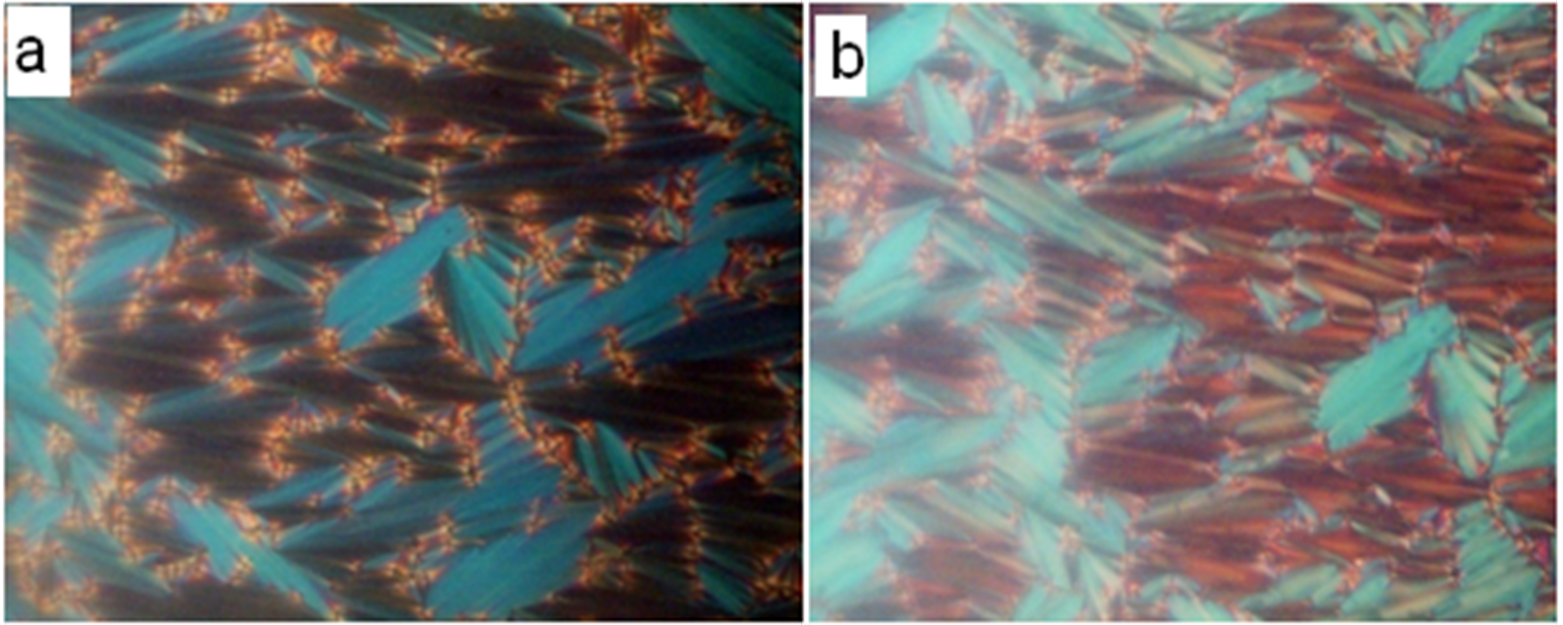
(a) Focal conic fan texture exhibited by **13ar** (Ph.Ox.C*C_10_) at 120 °C. (b) Broken focal conic fan texture exhibited by **13br** (C_12_.Ox.C*C_10_) at 65 °C.

**Figure 2:**
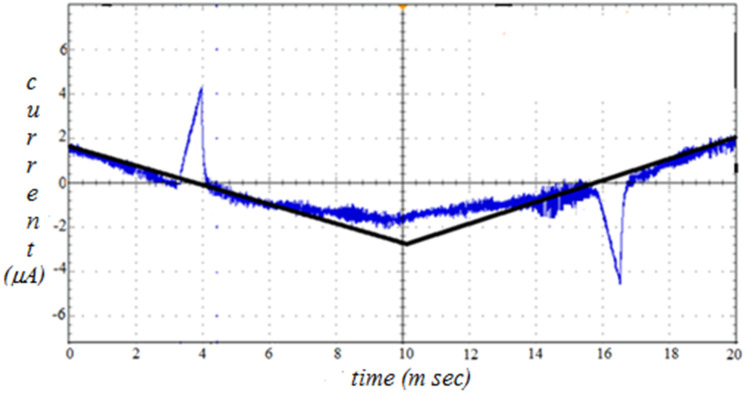
Polarization profile of **13br** (C_12_.Ox.C*C_10_) compound at 65 °C.

**Figure 3:**
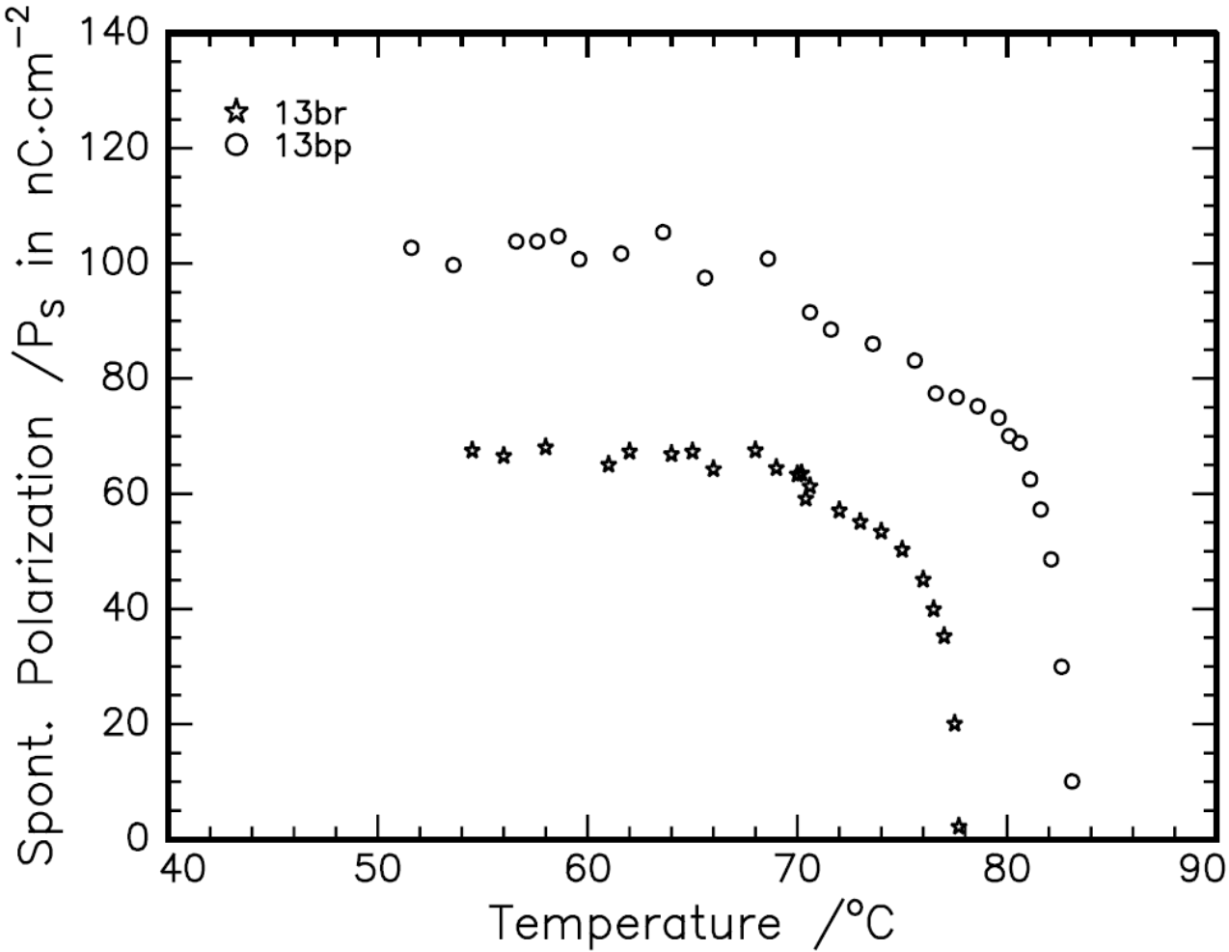
Temperature dependant spontaneous polarisation in **13bp** and **13br**.

The mesomorphism exhibited by the compounds is characterized by a polarizing optical microscope (POM) equipped with a hot stage. The sample is sandwiched between a glass plate and a cover slip and is subjected to heating followed by cooling scans. The observed textural changes through POM under crossed polarizers are simultaneously recorded. On heating the sample under crossed polarizers, the solid sample is melted and a focal conic fan texture is observed. On further heating the focal conic fan texture disappeared and transformed into an isotropic liquid. This observation is noticed for both the **13a** and **13b** compound series. The phase observed both in heating and cooling cycles are called enantiotropic while that observed only in cooling is termed monotropic. On cooling the isotropic liquid of the sample, batonnets are formed, which are found to grow and coalesce to form a focal conic fan texture. It is also observed that the sample exhibits a pseudo-isotropic texture in the homeotropic regions simultaneously. This texture is similar to the texture reported [38] for the calamitic SmA phase of *N*-(*p*-*n*-tridecyloxybenzylidene)-*p*-*n*-butylaniline. This confirms that the phase is an orthogonal smectic-A (SmA) phase. The optical texture of the SmA phase exhibited by **13ar** (Ph.Ox.C^*^C_10_) is given in Figure 1a as a representative of the series. Similar textural changes are observed in other compounds of the **13a** (Ph.Ox.C^*^C*_n_*) and the **13b** (C_12_.Ox.C^*^C*_n_*) series. The compounds of the **13a** (Ph.Ox.C*C*_n_*) series exhibiting a SmA phase are found to get transformed into crystal phase upon further cooling. Since the SmA phase is observed in both heating and cooling cycles, it is an enantiotropic in both the **13a** and **13b** series. But, in the compounds of the **13b** (C_12_.Ox.C^*^C*_n_*) series during heating, the sample is melted and exhibiting focal conic fan texture. On further heating it is transformed into an isotropic liquid. During cooling, the isotropic phase is found to transform into the SmA phase. Interestingly, the focal conic fan texture of the SmA phase is found to be transformed into a broken focal conic fan texture on further cooling and the areas with pseudo-isotropic texture (in homeotropically configured regions) are found to develop schlieren threads. This simultaneous observation of threads and broken focal conic fan texture infers [39] that the LC phase is a tilted smectic phase. This texture is found to be similar to the texture reported [40] for the polar smectic-C (SmC*) phase of octyl 4-(4-(3-(4'-(undec-10-en-1-ylcarbonyloxy)biphenyl-4-carbonyloxy)benzoyloxy)benzoyloxy)benzoate. The same textural observations are noticed in all the compounds of the **13b** series. The broken focal conic fan texture is observed only in cooling cycle and hence it is a monotropic in this **13b** series of compounds. It is also noticed that when the tilted phase is subjected to an input triangular ac wave (20 Hz, 10 Vp-p bias field) (Figure 2), the smectic domains are started to switch and the output wave is found to develop a peak. The area under the peak is found to increase with decreasing the temperature. This observation reflects on the temperature-dependent spontaneous polarisation (P_S_) in the tilted SmC* phase. The maximum spontaneous polarisation of about 65 nC/cm^2^ and 100 nC/cm^2^ are observed in **13bp** (C_12_.Ox.C^*^C_10_) and **13br**, respectively. The temperature dependent P_S_ of **13bp** and **13br** are given in Figure 3 as representatives of the series. Hence, the tilted phase is confirmed as chiral SmC phase, i.e., SmC*. The optical microscopic texture of SmC* exhibited by **13br** (C_12_.Ox.C^*^C_10_) at 65 °C is given in Figure 1b as a representative case of the series. It is noticed that the compounds are transformed into a crystalline phase on further cooling from their SmC* phase. All the compounds in the **13b** (C_12_.Ox.C^*^C*_n_*) series are found to exhibit a similar sequence of phase transitions, i.e., Iso. – SmA – SmC* – Cryst. Hence, the LC phase variance exhibited by the **13a** and **13b** series is confirmed as SmA and SmA, SmC*, respectively.

**Figure 4:**
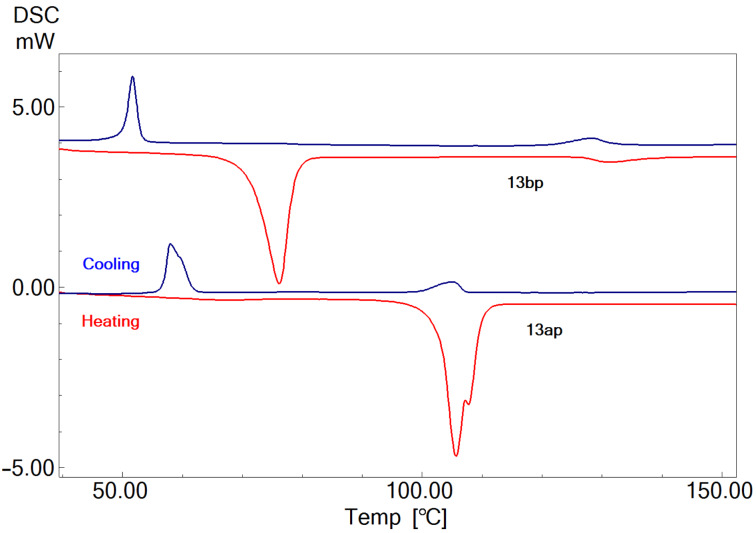
DSC thermograms of **13ap** and **13bp**.

The phase transition studies of all the compounds, i.e., the **13a** (PhOx.C^*^C*_n_*) and the **13b** (C_12_.Ox.C^*^C*_n_*) series are carried out by a Shimadzu (DSC-60) differential scanning calorimeter (DSC). The heating and cooling rates applied for the thermal studies are 10 °C/min. The thermograms of **13ap** and **13bp** are given in Figure 4 as representatives of the series. Compound **13ap** in heating cycle shows a sharp peak at 105.7 °C with a shoulder at 107.8 °C (i.e., not well resolved) corresponding to melting and isotropic liquid transitions, respectively. But the peaks are well resolved in cooling cycle.

In case of **13bp**, the peaks at 76.1 °C and 131.4 °C are due to melting and isotropic liquid transitions, respectively. The SmC* phase is not noticed in POM studies during the heating cycle, but the characteristic phase textural changes are noticed in the cooling cycle, i.e., it is monotropic in nature. This is observed in other compounds of the **13b** series too. From the POM observations, it is expected that compound **13bp** should show a peak corresponding to SmA – SmC* during the cooling cycle in thermal studies along with the Iso. – SmA and crystallisation peaks. But, the SmA – SmC* transition is noticed as a very small peak (not a prominent) with a very low enthalpy change value. In anticipation of the evolving SmA – SmC* transition, the sample is subjected to heating and cooling at low scan rates, i.e., at 5 °C/min. Even at this low scan rate, the SmA – SmC* transition is not evolved clearly in the **13b** series. It is expected that the SmA – SmC* transition is of a weak first order or second order transition due to the changeover of the orthogonal to tilted phase structure with less tilt (about the normal). The minimal ∆*H* values noticed can be attributed to the second order phase transition in the **13b** series. The thermograms obtained during the thermal studies at 5 °C/min are given in Supporting Information File 1. It is noticed that the mesomorphic thermal range is almost the same in both cases. The data of transition temperatures (*T*_C_), the enthalpy changes (∆*H*) across the transitions and entropy changes (∆*S*) of **13a** (Ph.Ox.C^*^C*_n_*) and **13b** (C_12_.Ox.C^*^C*_n_*) are presented in Table 1 and Table 2, respectively.

**Table 1:** Phase transition temperatures (°C), enthalpy changes (kJ/mol) and entropy changes (J/mol/K) of the **13a** (Ph.Ox.C*C*_n_*) series.

Compound	Phase transition	Phase transition temperatures	Δ*H*	Δ*S*	(Δ*Τ*)_LC_

**13ap**	Cryst. – SmA	105.7			
	SmA – Iso.	107.8^a^	28.52^b^	75.3	
	Iso. – SmA	105.3	1.88	4.97	
	SmA – Cryst.	58.0	16.09	48.6	47.3
**13aq**	Cryst. – SmA	106.4			
	SmA – Iso.	108.5^a^	59.58^b^	157.04	
	Iso. – SmA	103.4	3.87	10.28	
	SmA – Cryst.	58.1	29.14	88.01	45.3
**13ar**	Cryst. – SmA	98.9	19.55	52.57	
	SmA – Iso.	104.6	1.20	3.18	
	Iso. – SmA	109.0	1.85	4.84	
	SmA – Cryst.	62.0	17.32	51.70	47.0
**13as**	Cryst. – SmA	95.1	22.67	61.59	
	SmA – Iso.	106.0	2.39	6.31	
	Iso. – SmA	107.6	2.43	6.38	
	SmA – Cryst.	55.9	11.77	35.78	51.7

^a^Not well resolved, ^b^combined enthalpy change.

**Table 2:** Phase transition temperatures (°C), enthalpy changes (kJ/mol) and entropy changes (J/mol/K) of the **13b** (C_12_Ox.C*C*_n_*) series.

Compound	Phase transition	Phase transition temperatures	Δ*H*	Δ*S*	(Δ*Τ*)_LC_	(Δ*Τ*)_orthog_	(Δ*Τ*)_tilted_

**13bp**	Cryst. – SmA	76.1	39.42	112.9			
	SmA – Iso.	131.3	2.34	5.8			
	Iso. – SmA	128.2	2.57	6.4			
	SmA – SmC*	83.6	0.34	0.95			
	SmC* – Cryst.	51.7	25.37	78.1	76.5	44.6	31.9
**13bq**	Cryst. – SmA	76.4	40.70	116.5			
	SmA – Iso.	133.7	2.51	6.2			
	Iso. – SmA	128.7	2.76	6.9			
	SmA – SmC*	79.4	0.28	0.79			
	SmC* – Cryst.	54.0	11.92	36.4	74.7	49.3	25.4
**13br**	Cryst. – SmA	79.0	56.31	160.0			
	SmA – Iso.	128.2	2.44	6.1			
	Iso. – SmA	127.2	3.40	8.5			
	SmA – SmC*	77.7	0.54	1.5			
	SmC* – Cryst.	54.6	30.82	94.1	72.6	49.5	23.1
**13bs**	Cryst. – SmA	80.7	48.06	135.9			
	SmA – Iso.	137.1	2.50	6.1			
	Iso. – SmA	137.0	2.51	6.1			
	SmA – SmC*	75.0	0.21	0.60			
	SmC* – Cryst.	47.8	28.45	88.7	89.2	62.0	27.2

**Figure 5:**
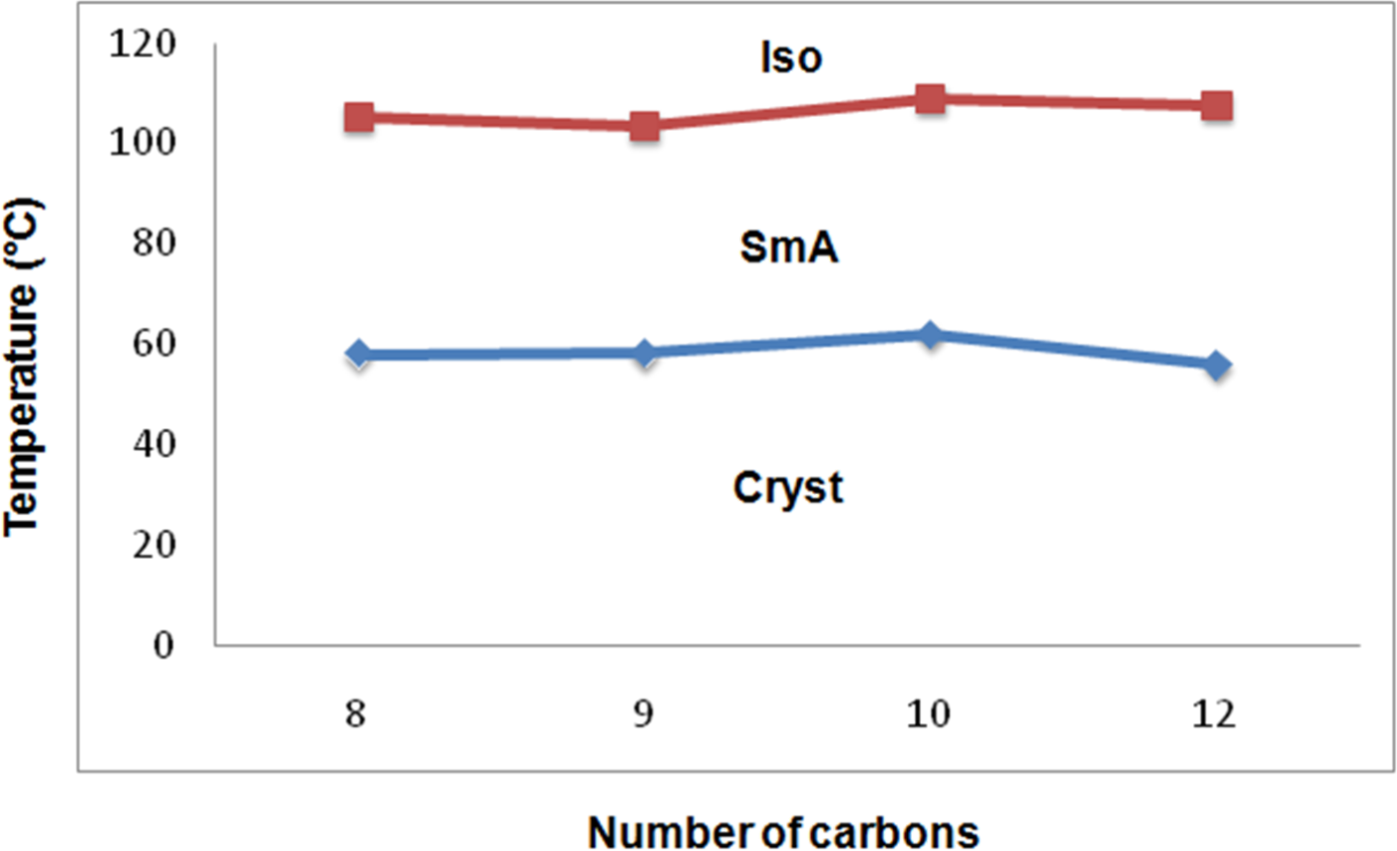
Phase diagram of the **13a** (Ph.Ox.C*C*_n_*) series.

**Figure 6:**
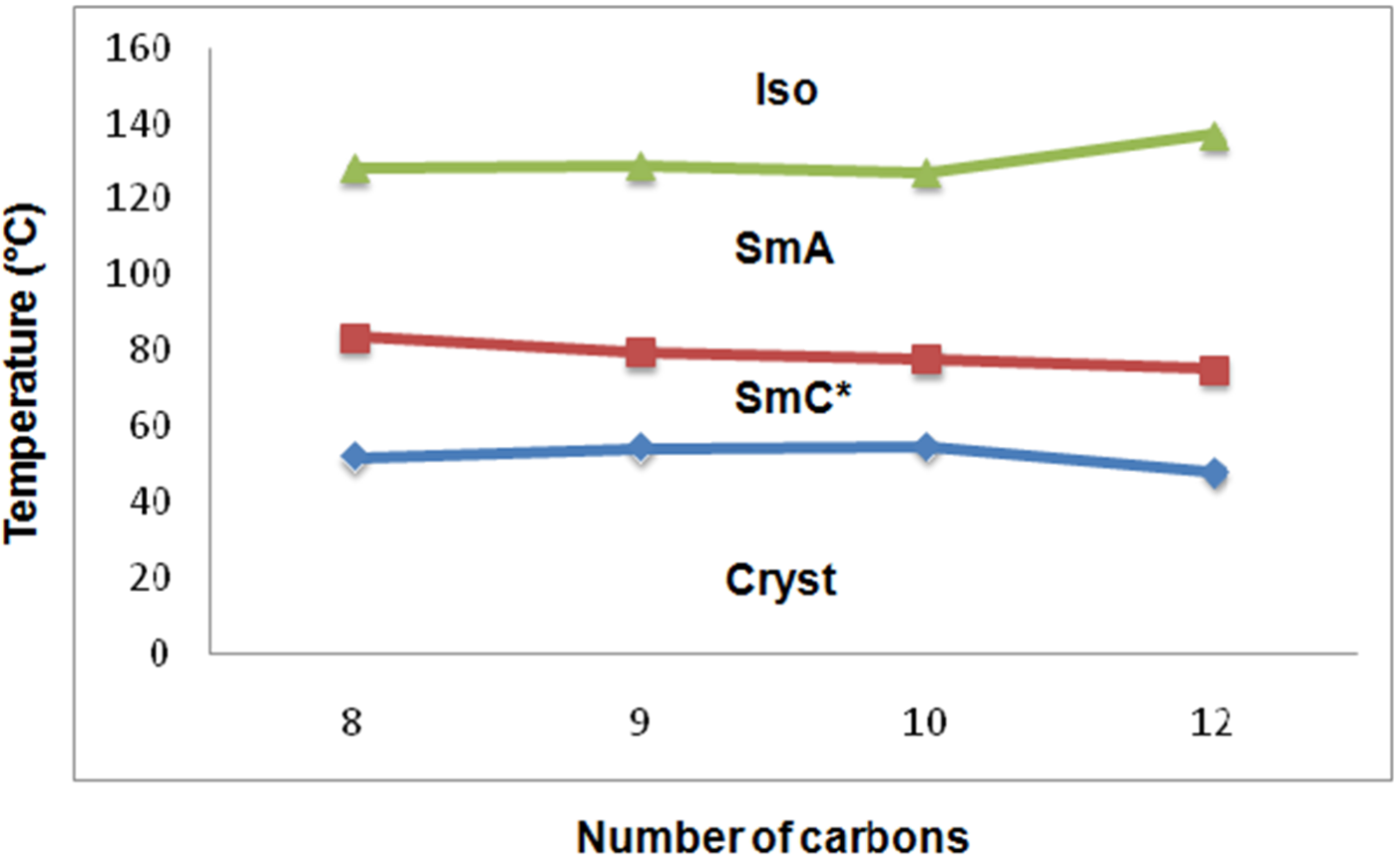
Phase diagram of the **13b** (C_12_.Ox.C*C*_n_*) series.

The phase diagrams are constructed with DSC cooling cycle data of the **13a** (Ph.Ox.C^*^C*_n_*) and the **13b** (C_12_.Ox.C^*^C*_n_*) series and are presented in Figure 5 and Figure 6, respectively. It is realized that the compounds of the **13a** (Ph.Ox.C^*^C*_n_*) series contain a rigid phenyl moiety on one side and a flexible alkyl chain on the other side of the oxadiazole central core. They are found to exhibit a 1D orthogonal SmA mesophase only. Further, the mesomorphic thermal range is not appreciably changing with increasing the chain length (Figure 5). The average mesomorphic thermal range of the compounds in this series (**13a**) is found to be about 48 °C.

On the other hand, the **13b** (C_12_.Ox.C^*^C*_n_*) series comprises of the flexible alkyl end chains on both sides of the central oxadiazole core. This series of compounds are found to exhibit both orthogonal and tilted phases viz., SmA and SmC*. However, the mesomorphic thermal ranges of the compounds are found to be higher than the other series (**13a**) of the present study.

We can recall [41–43] that the mesomorphic thermal ranges and the stabilities of the compounds are affected by the polarity, polarizability and intermolecular interactions between the molecules. Comparison between the orthogonal mesomorphic thermal ranges of both of the series, the series of the compounds with flexible chains on both sides, i.e., **13b** are found to exhibit a higher mesomorphic thermal range than the other series, i.e., **13a** with phenyl end group. The large mesomorphic thermal range in the **13b** (C_12_.Ox.C^*^C*_n_*) series are due to the higher number of methylene units in the alkyl end chain, which results in the higher surface area and higher London dispersion forces between the molecules. Interestingly, a tilted chiral SmC phase is also noticed in the **13b** (C_12_.Ox.C^*^C*_n_*) series along with the 1D orthogonal SmA phase. Due to the presence of long chains on both sides of the oxadiazole core, the molecules are argued to favour [44–46] the orientational disorder, which leads to the stabilization of tilt layered structures. The mesomorphic thermal range of the SmC* phase is found to be less than the SmA phase exhibited by both series. The average mesomorphic thermal range of an orthogonal SmA phase is almost the same in both series, i.e., (∆*T*)_SmA_ is about 48 °C and 51 °C in the **13a** and the **13b** series, respectively. The average mesomorphic thermal range of the tilted phase (SmC*) exhibited by the **13b** (C_12_.Ox.C^*^C*_n_*) series is found to be about 27 °C.

The mesomorphic thermal range is found to be effected marginally with increasing the end chain length in both series. The mesomorphic thermal range is found to increase greatly in the **13b** series possessing a chiral center and an aliphatic flexible end chain with 12 carbons (i.e., 89.2 °C) against the corresponding member of the **13a** series (51.7 °C). Both of the series seem to follow the odd-even effect [47] regarding clearing (isotropic) temperatures to infer the impact of axial polarizabilities. An overview of tilted and orthogonal LC phase abundance exhibited by the **13a** and **13b** series (Table 1 and Table 2) reveal that the later series with flexible end chain yielded approximately 56% of tilted phase structures and is argued due to the relative strength of orientational disorder in chiral LCs with flexible end chain on both sides.

It is noticed that the isotropic temperatures are higher for the **13b** (C_12_.Ox.C^*^C*_n_*) series than the other phenyl substituted **13a** homologs. Higher clearing temperatures are argued due to the chain geometry and close packing of the molecules in the **13b** (C_12_.Ox.C^*^C*_n_*) series. The compounds in the **13a** (Ph.Ox.C^*^C*_n_*) series consist of a rigid and bulky phenyl moiety on one end, which tends to keep the molecules away due to the repulsive forces. Also, the crystallization temperatures (i.e., melting temperatures) of the **13a** (Ph.Ox.C^*^C*_n_*) series are found to be higher than those of the **13b** (C_12_.Ox.C^*^C*_n_*) series.

## Conclusion

Two novel series of ferroelectric liquid crystalline materials with 1,2,4-oxadiazole central core are synthesized and characterized. The phenyl moiety at the C-5 position of the oxadiazole core is converted into a biphenyl moiety by Suzuki coupling reaction. Both the series of compounds have the same molecular skeleton except the end group on one side of the central core. The series with the bulky phenyl end group on one side of the oxadiazole core is found to exhibit a 1D orthogonal SmA mesophase only whereas the other series with flexible alkyl end chain are found to exhibit chiral SmC phase along with the SmA mesophase. The compounds with flexible end chains on both sides (**13b** or C_12_.Ox.C^*^C*_n_*) of the oxadiazole core are found to exhibit a higher mesomorphic thermal range than the series with a phenyl end group. The compounds of the **13b** (C_12_.Ox.C^*^C*_n_*) series are also exhibiting the ferroelectric switching in their tilted SmC phase.

## Experimental

The starting materials required in the synthesis are procured from Sigma-Aldrich, Spectrochem, and Alfa-Aesar and are used without further purification. AR grade solvents are used for the synthesis. The products obtained in the various stages of synthesis are purified by column chromatography with silica gel (230–400 mesh) as a stationary phase and an appropriate solvent system as mobile phase. FTIR spectra (cm^−1^) are recorded on a Perkin-Elmer 283 spectrometer by the KBr pellet method. ^1^H and ^13^C NMR spectra are recorded on Bruker NMR (400 MHz) spectrometers using CDCl_3_ as solvent. Elemental analyses are carried out using an Elementar vario MICRO cube. Polarization exhibited by tilted LC phase structures is determined by field reversal method.

## Supporting Information

The experimental procedure for the synthesis of **13ap–13as** and **13bp–13bs** with the intermediate steps involved and the spectral characterization are given in Supporting Information File 1.

File 1 Experimental and analytical data.
