# Supplementary material for: Novel biphenyl-substituted 1,2,4-oxadiazole ferroelectric liquid crystals: synthesis and characterization
Source: Beilstein J Org Chem. 2015 Feb 11;11:233–41. doi: 10.3762/bjoc.11.26 (PMC4361968; doi:10.3762/bjoc.11.26)
Supplement: File 1 — Experimental and analytical data. [file Beilstein_J_Org_Chem-11-233-s001.pdf]

Supporting Information

for

Novel biphenyl-substituted 1,2,4-oxadiazole ferroelectric liquid crystals: synthesis and characterization

Mahabaleshwara Subrao[1,2], Dakshina Murthy Potukuchi[3], Girish Sharada Ramachandra[1], Poornima Bhagavath[1], Sangeetha G. Bhat[1] and Srinivasulu Maddasani[1]*

Address: [1]Department of Chemistry, Manipal Institute of Technology, Manipal University, Manipal - 576 104, India, [2]Syngene International Ltd.,Biocon Park, Bommasandra, Bangalore - 560 099 and [3]Department of Physics, University College of Engineering, Jawaharlal Nehru Technological University: Kakinada, Kakinada - 533 003, India.

Email: Srinivasulu Maddasani* - s.maddasani@manipal.edu

*Corresponding author

Experimental and analytical data

Procedure for the synthesis of benzyl 4-cyanobenzoate (2a)

To a stirred solution of 4-cyanobenzoic acid (5 g, 34 mmol) in dry DMF, K_2CO_3 (7.03g, 51 mmol) is added. Then benzyl bromide (7.0 g, 41 mmol) is added drop wise and the reaction mixture is heated at 80 °C for 12 h. The reaction mixture is quenched in ice water and the oily product is extracted with ethylacetate. It is washed with brine followed by water. The organic layer is dried over anhydrous sodium sulfate and concentrated. The crude product is purified by column chromatography, using silica gel and ethylacetate/hexane (2:98). After evaporating the solvent, a white solid is obtained. The yield of the product is 78%. The ^{1}H NMR (300 MHz, $CDCl_3$): δ (ppm) 5.39 (s, 2H), 7.37-7.47 (m, 5H), 7.74 (d, J = 6.81 Hz, 2H), 8.17 (d, J = 6.75 Hz, 2H). Elemental analysis: calculated for $C_{15}H_{11}NO_2$ (237.25) C 75.94, H 4.67, N 5.90. Found: C 75.83, H 4.61, N 5.95.

Procedure for the synthesis of dodecyl 4-cyanobenzoate (2b)

To a stirred solution of 4-cyanobenzoic acid (5 g, 34.0 mmol) in dry dichloromethane, DCC (10.51 g, 51.0 mmol) and a catalytic amount of DMAP are added under nitrogen atmosphere, followed by the addition of 1-dodecanol (7.51 g, 40.8 mmol). The reaction mixture is stirred at rt for 16 h. The urea (by product) is filtered and the filtrate was concentrated to get the crude product. The crude product is purified by column chromatography using silica gel and EtOAc:petroleum ether (2:98). A white solid product is obtained with a yield of 70%. The ^{1}H NMR (300 MHz, $CDCl_3$): δ (ppm) 0.89 (t, J = 6.56 Hz, 3H), 1.26- 1.37 (m, 18H), 1.73 - 1.82 (m, 2H), 4.35 (t, J =6.66 Hz, 2H), 6.75 (d, J = 6.90 Hz, 2H), 8.14 (d, J = 6.81 Hz, 2H). Elemental analysis: calculated for $C_{20}H_{29}NO_2$ (315.45) C 76.15, H 9.27, N 4.440. Found: C 76.08, H 9.21, N 4.47.

General procedure for the synthesis of benzyl/dodecyl 4-(*N*'-hydroxycarbamimidoyl)-benzoate (3a, 3b)

To a stirred solution of **2a/2b** (25.0 mmol) in ethanol, hydroxylamine hydrochloride (28.0 mmol) followed by sodium hydroxide (28.0 mmol) solution are added and refluxed for 3 h. The reaction mixture is cooled in a freezer for 24 h., a white crystalline product is obtained. The product is filtered, recrystallized with ethanol and dried.

Benzyl 4-(N'-hydroxycarbamimidoyl)benzoate **(3a):** Yield 80%, ^{1}H NMR (400 MHz, $CDCl_3$) δ (ppm): 4.99 (s, Br 1H), 5.38 (s, 2H), 7.34 - 7.42 (m, 3H), 7.46 (d, J = 7.20 Hz, 2H), 7.70 (d, J = 8.28 Hz, 2H), 8.10 (d, J = 8.24 Hz, 2H). Elemental analysis: calculated for $C_{15}H_{14}N_2O_3$ (270.28) C 66.66, H 5.22, N 10.36. Found: C 66.61, H 5.19, N 10.42.

Dodecyl 4-(N'-hydroxycarbamimidoyl)benzoate **(3b):** Yield 83%, ^{1}H NMR (300 MHz, $CDCl_3$) δ (ppm): 0.88 (t, J = 6.87 Hz, 3H), 1.26 - 1.41 (m, 18H), 1.72 - 1.79 (m, 2H), 4.32 (t, J = 6.63 Hz, 2H), 5.12 (s, Br 2H), 7.69 (d, J = 8.4 Hz, 2H), 8.06 (d, J = 8.4 Hz, 2H). Elemental analysis: calculated for $C_{20}H_{32}N_2O_3$ (348.48) C 68.93, H 9.26, N 8.04. Found: C 68.87, H 9.22, N 8.12%.

General procedure for the synthesis of benzyl/dodecyl 4-(5-(4-bromophenyl)-1,2,4-oxadiazol-3-yl)-benzoate (5a, 5b)

The product **3a/3b** (3.7 mmol) is dissolved in dry pyridine. It is cooled to 0 °C followed by the addition of 4-bromobenzoyl chloride, **4** (3.7 mmol). The mixture is refluxed for 5 h. and then cooled to rt and poured into ice cold water. The crude product is filtered and recrystallized with ethanol.

Benzyl 4-(5-(4-bromophenyl)-1,2,4-oxadiazol-3-yl)benzoate **(5a):** Yield 62%, ^{1}H NMR (400 MHz, $CDCl_3$): δ (ppm) 5.41 (s, 2H), 7.37 - 7.42 (m, 3H), 7.48 (d, *J* = 7.12, 2H), 8.72 (d, *J* = 8.40 Hz, 2H), 8.10 (d, *J* = 8.44 Hz, 2H), 8.20 - 8.26 (m, 4H). Elemental Analysis: calculated for $C_{22}H_{14}BrN_2O_3$ (435.27) C 60.71, H 3.47. N 6.44, Found: C 60.52, H 3.38, N 6.37%.

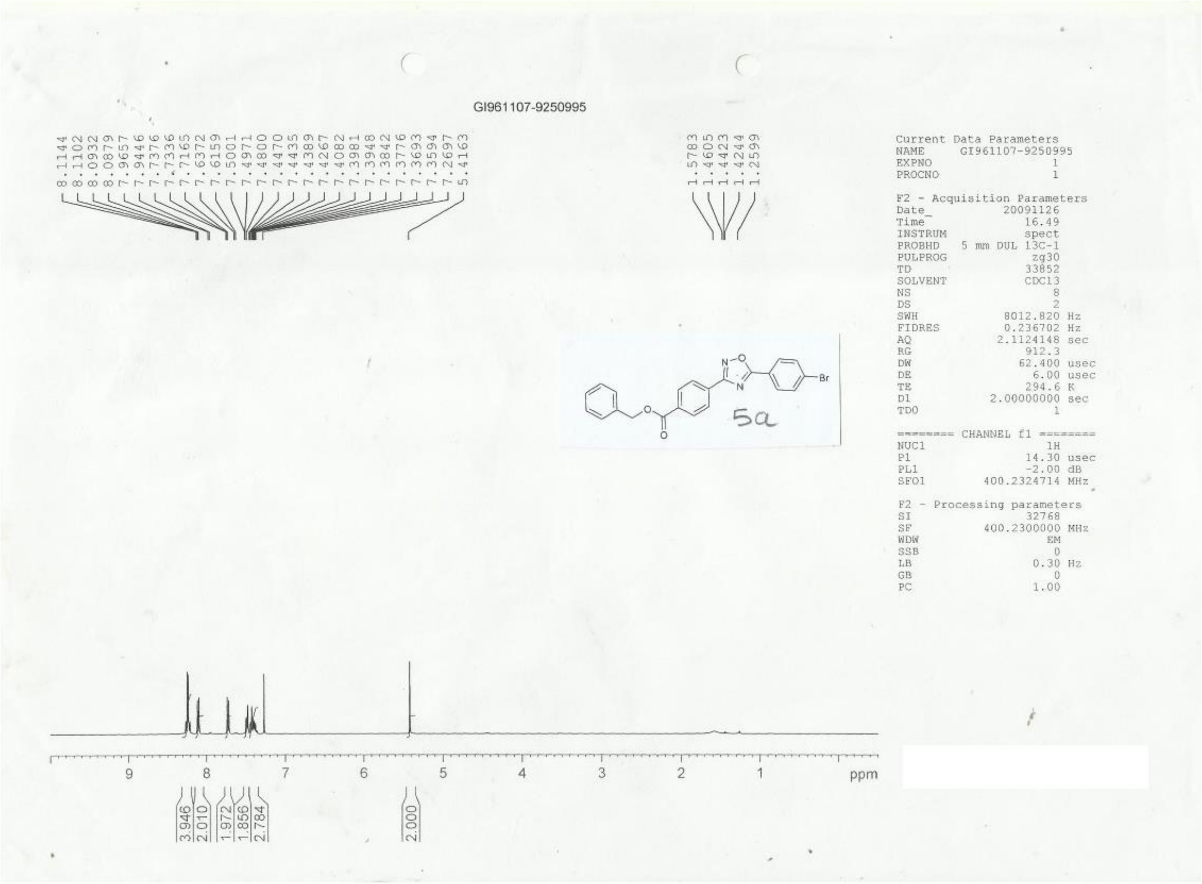

Fig. 1: ^{1}H NMR of **5a**

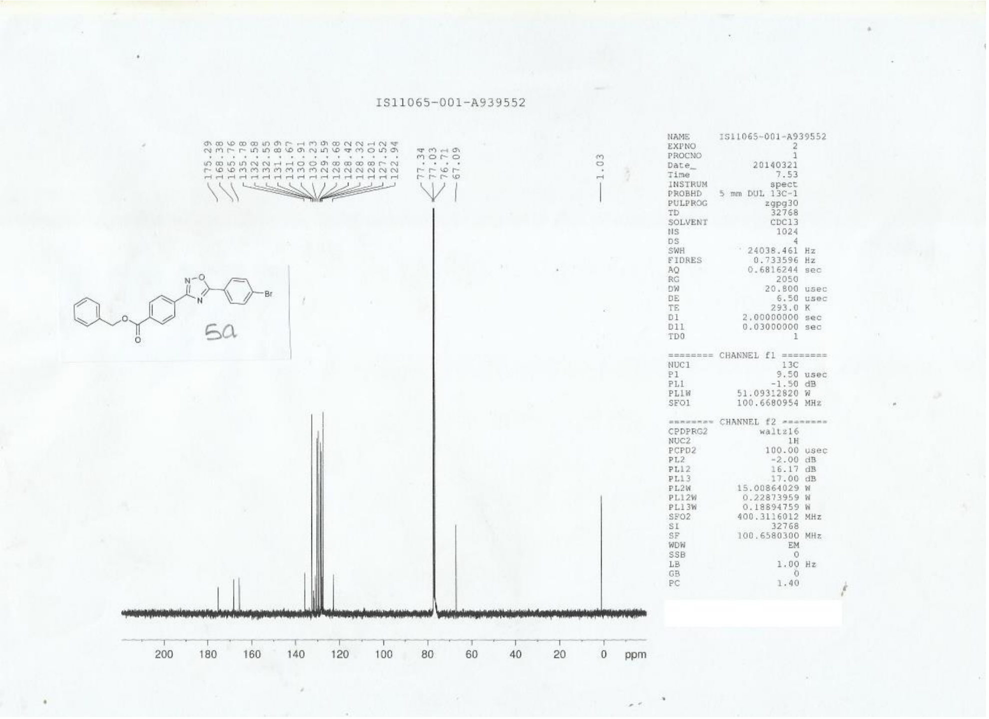

Fig. 2: ^{13}C NMR of **5a**

Dodecyl 4-(5-(4-bromophenyl)-1,2,4-oxadiazol-3-yl)benzoate **(5b):** Yield 73%, ^{1}H NMR (400 MHz, $CDCl_3$) δ (ppm): 0.88 (t, *J* = 6.78 Hz, 3H), 1.42 - 1.48 (m, 18H), 1.75 - 1.85 (m, 2H), 4.36 (t, *J* = 6.68 Hz, 2H), 7.72 (d, *J* = 8.6 Hz, 2H), 8.10 (d, *J* = 6.78 Hz, 2H), 8.18 (d, *J* = 8.56 Hz, 2H), 8.24 (d, *J* = 6.8 Hz, 2H). Elemental analysis: calculated for $C_{27}H_{33}BrN_2O_3$ (513.47) C 63.16, H 6.48, N 5.46. Found: C 63.10, H 6.43, N 5.43%.

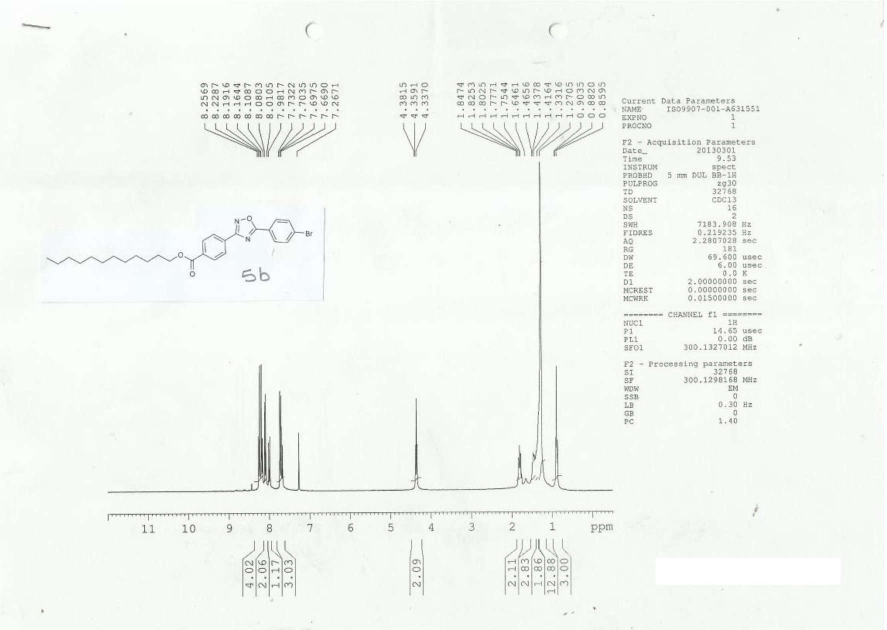

Fig. 3: ^{1}H NMR of **5b**

General procedure for the synthesis of boronate esters, 6a/6b

To a stirred solution of **5a/5b** (2.3 mmol) in 1,4-dioxane, bis(pinacolato)diborane (Pin_2B_2) (3.4 mmol) and potassium acetate (10.3 mmol) are added. It is stirred for 5 min. with continuous purging of nitrogen gas followed by the addition of catalyst, Pd(dppf)Cl_2. The resulting reaction mixture is stirred at 100 °C for 16 h then it is cooled. The solvent is removed by distillation; the crude product is extracted with EtoAc and is filtered through celite. It is purified by column chromatography using silica gel and EtoAc:hexane. The products are off white solids.

Benzyl 4-(5-(4-(4,4,5,5-tetramethyl-1,3,2-dioxaborolan-2-yl)phenyl)-1,2,4-oxadiazol-3-yl)-benzoate **(6a):** Yield 59.1% ^{1}H NMR (300 MHz, $CDCl_3$), δ (ppm) 1.38 (s, 12H), 5.41 (s, 2H), 7.37 - 7.50 (m, 6H), 7.99 (d, *J* = 8.25 Hz, 2H), 8.20 - 8.28 (m, 5H). Elemental Analysis: calculated for $C_{28}H_{27}BN_2O_5$ (482.33) C 69.72, H 5.64, N 5.81, Found: C 69.63, H 5.67, N 5.78%.

Dodecyl 4-(5-(4-(4,4,5,5-tetramethyl-1,3,2-dioxaborolan-2-yl)phenyl)-1,2,4-oxadiazol-3-yl)benzoate **(6b):** Yield 67.7%; ^{1}H NMR (400 MHz, $CDCl_3$) δ (ppm): 0.87 (t, *J* = 6.4 Hz, 3H), 1.26 - 1.28 (m, 18H), 1.39 (s,12H), 1.74 - 1.83 (m, 2H), 4.35 (t, *J* = 6.0 Hz, 2H), 8.00 (d, *J* = 7.6 Hz, 2H), 8.17 - 8.23 (m, 4H), 8.27 (d, *J* = 8.0 Hz, 2H); Elemental Analysis: calculated for $C_{33}H_{45}BN_2O_5$ (560.53) C 70.71, H 8.09, N 5.00, Found: C 70.45, H 7.92, N 4.94%.

Procedure for the synthesis of (*S*)-(+)-methyl-3-(benzyloxy)-2-methylpropanoate (8)

To a stirred hexane solution of **7** (20 g, 0.17 mol), benzyl 2,2,2-trichloroacetimidate (53.3 g, 0.21 mol) is added and stirred for 30 min. followed by CF_3SO_3H (2.5g, 0.017 mol). The obtained white solid is filtered out and the filtrate is purified by column chromatography. ^{1}H NMR (300 MHz $CDCl_3$), δ (ppm) 1.18 (d, *J* = 7.1Hz, 3H), 2.71 - 2.88 (m, 1H), 3.49 (dd, *J* = 5.92, 9.10 Hz, 1H), 3.66 (dd, *J* = 7.24, 9.12 Hz, 1H),3.69 (s, 3H), 4.52 (s, 2H), 7.25 - 7.35 (m, 5H); Elemental Analysis: calculated for $C_{12}H_{16}O_3$ (208.25), C 69.21, H 7.74; Found: C 69.28, H 7.83%

Procedure for synthesis of (*S*)-(+)-3-(benzyloxy)-2-methylpropanoic acid (9)

To a stirred solution of **8** (2.5 g, 12.0 mmol) in methanol and water mixture, LiOH (2.51 g, 60.0 mmol) is added at 0 °C. The reaction mixture is stirred at rt for 16 h and the methanol is distilled off. It is then quenched with ice cold water and acidified with 1.5 N HCl followed by the extraction with dichloromethane. The organic layer is washed with brine and then the solvent is removed by distillation. The product, **9** is obtained as colourless oil which

is dried over anhydrous sodium sulfate. The yield of the product is about 87%. ^{1}H NMR (300 MHz $CDCl_3$), δ (ppm) 1.06 (d, *J*= 7.04Hz, 3H), 2.60- 2.65 (m, 1H), 3.45 (dd, *J* = 5.8, 9.12 Hz, 1H), 3.56 (dd, *J*= 6.96, 9.12Hz, 1H), 4.46 (s, 2H), 7.27 - 7.36 (m, 5H), 12.18 (s, Br, 1H). Elemental Analysis: calculated for $C_{11}H_{14}O_3$ (194.22), C 68.02, H 7.27, Found C 67.96, H 7.20%

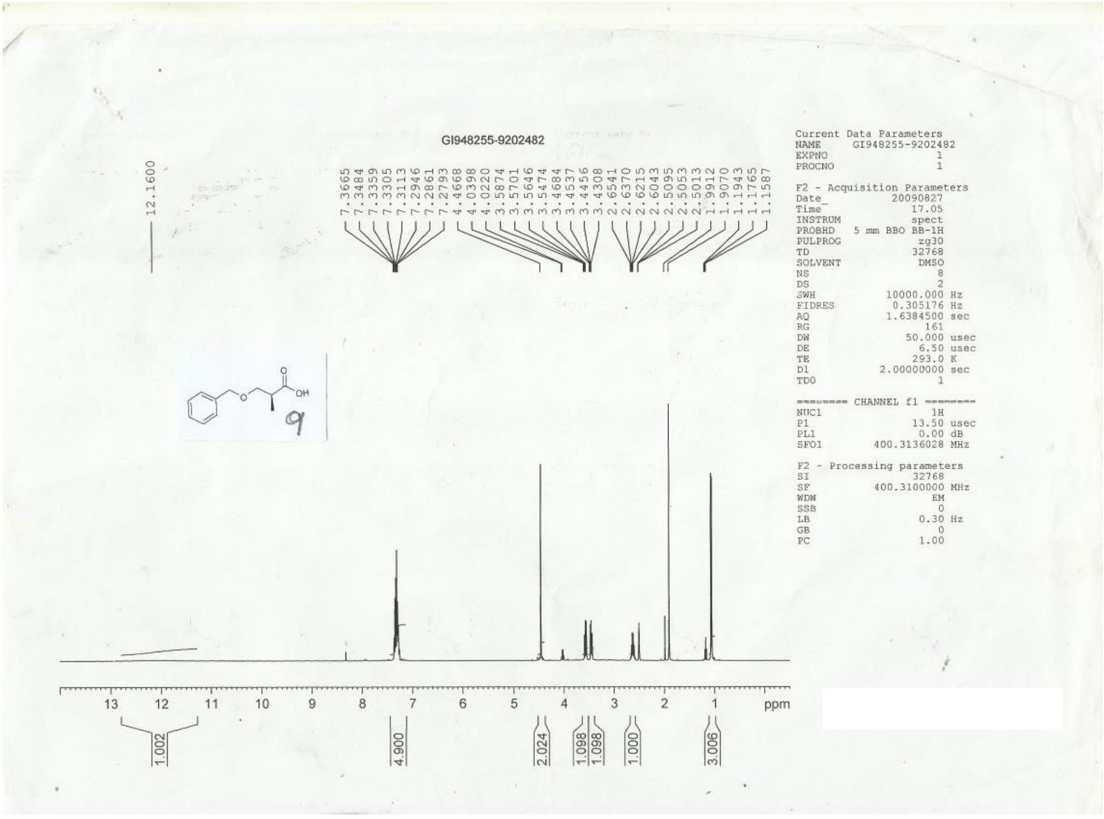

Fig. 4: ^{1}H NMR of **9**

General procedure for the synthesis of (*S*)-(+)-alkyl-3-(benzyloxy)-2-methylpropanoates, 10p–10s.

To a stirred solution of **9** (2.5 mmol) in dry dichloromethane is added DCC (2.9 mmol) and DMAP under nitrogen atmosphere, followed by the addition of long chain alcohol (3.8 mmol). After stirring the reaction mixture for 16 h, it is filtered and the filtrate is concentrated to get the corresponding crude products. The crude products are purified by column chromatography. The products, **10p–10s** are obtained as colourless oils.

(*S*)-(+)-*octyl 3-(benzyloxy)-2-methylpropanoate* **(10p):** Yield 70.5%; ^{1}H NMR (300 MHz, $CDCl_3$) δ (ppm) 0.88 (t, *J* = 6.12 Hz, 3H), 1.18 (d, *J* = 7.12 Hz, 3H), 1.27 - 1.40 (m, 10H), 1.59 - 1.64 (m, 2H), 2.74 - 2.81 (m, 1H), 3.50 (dd, *J* = 7.92, 12.12 Hz, 1H), 3.67 (dd, *J* = 9.68, 12.12 Hz, 1H), 4.09 (t, *J* = 6.69 Hz, 2H), 4.53 (s, 2H), 7.28 - 7.37 (m, 5H). Elemental Analysis: calculated for $C_{19}H_{30}O_3$ (306.43), C 74.47, H 9.87; Found C 74.28, H 9.79%.

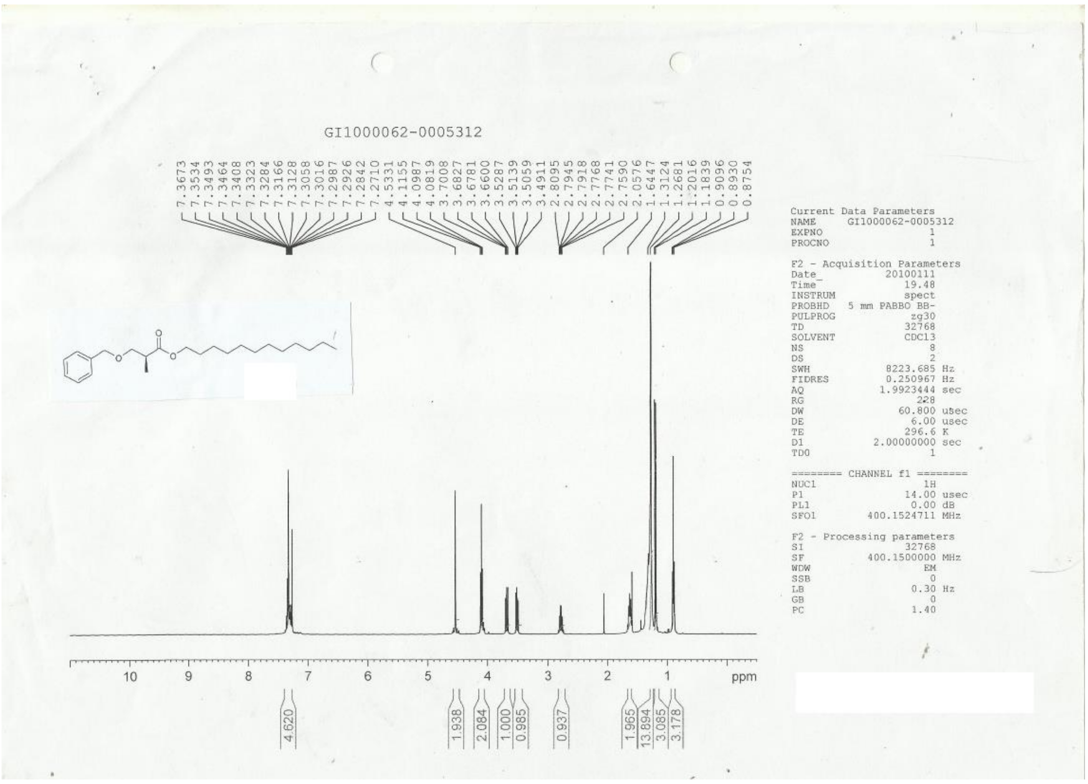

Fig. 5: ^{1}H NMR of **10p**

(*S*)-(+)-*nonyl-3-(benzyloxy)-2-methylpropanoate* **(10q):** Yield 71.3%; ^{1}H NMR (300 MHz, $CDCl_3$) δ (ppm)0.88 (t, *J* = 6.33 Hz, 3H), 1.18 (d, *J* = 7.08Hz, 3H), 1.28- 1.41(m, 12H), 1.57- 1.64(m, 2H), 2.74- 2.82(m, 1H), 3.51 (dd, *J* = 7.92, 12.10 Hz, 1H), 3.67 (dd, *J* = 9.64, 12.10Hz, 1H), 4.07(t, *J* = 6.70 Hz, 2H), 4.53(s, 2H), 7.26 - 7.34 (m, 5H).Elemental Analysis: calculated for $C_{20}H_{32}O_3$ (320.46), C 74.96, H 10.06; Found C 74.68, H 9.87%

(*S*)-(+)-*decyl-3-(benzyloxy)-2-methylpropanoate* **(10r):** Yield 70.5%; ^{1}H NMR (400 MHz, $CDCl_3$) δ (ppm)0.89 (t, *J* = 7.04 Hz, 3H),1.19 (d, *J*= 7.08Hz, 3H),1.26- 1.41(m, 14H),1.57- 1.64(m, 2H),2.75- 2.80(m, 1H),3.50 (dd, *J* = 5.96, 9.64 Hz, 1H), 3.66 (dd, *J* = 7.69, 9.64Hz, 1H),4.08(t, *J*= 6.72 Hz, 2H),4.53(s, 2H), 7.27 - 7.36 (m, 5H). Elemental Analysis: calculated for $C_{21}H_{34}O_3$ (334.49), C 75.41, H 10.25; Found: C 75.18, H 10.31%

(*S*)-(+)-*dodecyl-3-(benzyloxy)-2-methylpropanoate* **(10s):** Yield 72.7%; ^{1}H NMR (300 MHz, $CDCl_3$) δ (ppm) 0.88 (t, *J* = 6.93 Hz, 3H), 1.18 (d, *J*= 7.08Hz, 3H), 1.27 - 1.42 (m, 18H), 1.57 - 1.64 (m, 2H), 2.73 - 2.81 (m, 1H), 3.50 (dd, *J* = 5.92, 9.12 Hz, 1H), 3.67 (dd, *J* = 7.24, 9.08 Hz, 1H), 4.07 (t, *J*= 6.78 Hz, 2H), 4.52 (s, 2H), 7.26 - 7.34 (m, 5H), Elemental Analysis: calculated for $C_{23}H_{38}O_3$ (362.54), C 76.20, H 10.56; Found: C 76.21, H 10.48%

General procedure for the synthesis of (*S*)-(+)-alkyl-2-(hydroxymethyl)propanoates, 11p –11s

To a stirred solution of **10p–10s** (4.35 mmol) in EtOAc is added Pd/C (10%) at RT under nitrogen atmosphere. The reaction mixture is degassed and is stirred at RT for 4 h. under hydrogen atmosphere. The reaction mixture is filtered through celite and the filtrate is concentrated, yields colourless liquids.

(*S*)-(+)-o*ctyl-2-(hydroxymethyl)propanoate* **(11p):** Yield 93.8%; ^{1}H NMR (400 MHz, $CDCl_3$) δ (ppm) 0.89 (t, *J* = 6.96 Hz, 3H), 1.19 (d, *J* = 7.08Hz, 3H), 1.26 - 1.38 (m, 10H), 1.58 - 1.68 (m, 2H), 2.64 - 2.69 (m, 1H), 3.70 - 3.73 (m, 2H), 4.07 (t, *J* = 6.76 Hz, 2H); Elemental Analysis: calculated for $C_{12}H_{24}O_3$ (216.31), C 66.63, H 10.56; Found C 66.49, H 10.29%

(*S*)-(+)-*nonyl-2-(hydroxymethyl)propanoate* **(11q):** Yield 92.5%; ^{1}H NMR (400 MHz, $CDCl_3$) δ (ppm) 0.88 (t, *J* = 7. Hz, 3H), 1.18 (d, *J* = 7.12 Hz, 3H), 1.27 - 1.39 (m, 12H), 1.60 - 1.69 (m, 2H), 2.66 - 2.69 (m, 1H), 3.71 - 3.74 (m, 2H), 4.08 (t, *J* = 6.78 Hz, 2H); Elemental Analysis: calculated for $C_{13}H_{26}O_3$ (230.34), C 67.79, H 11.38; Found C67.59, H 11.26%

(*S*)-(+)-*decyl-2-(hydroxymethyl)propanoate* **(11r):** Yield 95.1%; ^{1}H NMR (400 MHz, $CDCl_3$) 0.89 (t, *J* = 6.98 Hz, 3H), 1.18 (d, *J*= 7.12 Hz, 3H), 1.25 - 1.38 (m, 14H), 1.57 - 1.68 (m, 2H), 2.64 - 2.68 (m, 1H), 3.70 - 3.73 (m, 2H), 4.08 (t, *J* = 6.76 Hz, 2H); Elemental Analysis: calculated for $C_{14}H_{28}O_3$ (244.37), C 68.81, H 11.55; Found C 68.71, H 11.65%

(*S*)-(+)-*dodecyl-2-(hydroxymethyl)propanoate* **(11s):** Yield 93.1%; ^{1}H NMR (400 MHz, $CDCl_3$) δ (ppm) 0.89 (t, *J* = 7.02 Hz, 3H), 1.18 (d, *J*= 7.12 Hz, 3H), 1.25- 1.38(m, 18H), 1.59- 1.68(m, 2H), 2.66- 2.68 (m, 1H), 3.70 - 3.74 (m, 2H), 4.07 (t, *J* = 6.74 Hz, 2H); Elemental Analysis: calculated for $C_{16}H_{32}O_3$ (272.42), C 70.54, H 11.84; Found C: 70.48, H:11.94%.

General procedure for the synthesis of (S)-(+)-2-((alkyloxy)carbonyl)propyl-4-bromobenzoates, 12p-12s

To a stirred solution of **11p–11s** (2.4 mmol) in dry dichloromethane are added DCC (2.7 mmol) and DMAP (0.24 mmol) under nitrogen atmosphere, followed by the addition of and *n*-alkylalcohol (2.4 mmol). After stirring the reaction mixture for 16 h., it is filtered and the filtrate is concentrated to get the corresponding crude products. The crude products are purified by column chromatography. The products, **12p–12s** are low-melting solids.

(*S*)-(+)-*2-((octyloxy)carbonyl)propyl-4-bromobenzoate* **(12p):** Yield 67.7%; ^{1}H NMR (400 MHz, $CDCl_3$) δ (ppm) 0.88 (t, *J* = 7.18 Hz, 3H), 1.12 - 1.27 (m, 10H), 1.28 (d, *J* = 7.10 Hz, 3H), 1.59 - 1.63 (m, 2H), 2.92 - 2.94 (m, 1H), 4.11 (t, *J* = 6.62 Hz, 2H), 4.42 - 4.47 (m, 2H), 7.57 (d, *J* = 6.82 Hz, 2H), 7.86 (d, *J* = 7.66 Hz, 2H); Elemental Analysis: calculated for $C_{19}H_{27}BrO_4$ (399.31), C 57.15, H 6.82; Found: C 57.25, H 6.72%.

(*S*)-(+)-*2-((nonyloxy)carbonyl)propyl-4-bromobenzoate* **(12q):** Yield 61.1%; ^{1}H NMR (400 MHz, $CDCl_3$) δ (ppm) 0.87 (t, *J* = 7.10 Hz, 3H), 1.11 - 1.24 (m, 12H), 1.29 (d, *J* = 7.12 Hz, 3H), 1.57 - 1.62 (m, 2H), 2.92 - 2.95 (m, 1H), 4.12 (t, *J* = 6.64 Hz, 2H), 4.38 - 4.48 (m, 2H), 7.58 (d, *J* = 8.54 Hz, 2H), 7.87 (d, *J* = 8.76 Hz, 2H); Elemental Analysis: calculated for $C_{20}H_{29}BrO_4$ (413.34), C 58.11, H 7.07; Found: C 58.19, H 6.87%.

(*S*)-(+)-*2-((decyloxy)carbonyl)propyl-4-bromobenzoate* **(12r):** Yield 64.8%; ^{1}H NMR (400 MHz, $CDCl_3$) δ (ppm) 0.88 (t, *J* = 7.08 Hz, 3H), 1.10 - 1.25 (m, 14H), 1.28 (d, *J*= 7.16 Hz, 3H), 1.56 - 1.63 (m, 2H), 2.91 - 2.96 (m, 1H), 4.11 (t, *J* = 6.64 Hz, 2H), 4.39 - 4.49 (m, 2H), 7.57 (d, *J* = 8.56 Hz, 2H), 7.86 (d, *J* = 8.78 Hz, 2H); Elemental Analysis: calculated for $C_{21}H_{31}BrO_4$ (427.37), C 59.02, H 7.31; Found: C 59.10, H 7.21%.

(*S*)-(+)-*2-((dodecyloxy)carbonyl)propyl-4-bromobenzoate* **(12s):** Yield 66.3%; ^{1}H NMR (400 MHz, $CDCl_3$) δ (ppm) 0.89 (t, *J* = 7.04 Hz, 3H),1.11 - 1.26 (m, 18H), 1.29 (d, *J*= 7.08 Hz, 3H), 1.58 - 1.62 (m, 2H), 2.93- 2.95(m, 1H), 4.12(t, *J* = 6.60 Hz, 2H), 4.43- 4.47(m, 2H),7.58 (d, *J* = 6.84 Hz, 2H), 7.87 (d, *J* = 7.66 Hz, 2H); Elemental Analysis: calculated for $C_{23}H_{35}BrO_4$ (455.42), C 60.66, H 7.75; Found: C 60.52, H 7.42%.

General procedure for the synthesis of 13ap–13as (Ph.Ox.C*C$_n$) and 13bp–13bs (C_{12}Ox.C*C$_n$)

To a stirred solution of **12p-12s** (1.20 mmol) and **6a/6b** (1.00 mmol) in 1,2-dimethoxyethane, aq. Na_2CO_3 (2.2 mmol) is added under nitrogen purging for 15 min. followed by the addition of $Pd(pph_3)_2Cl_2$ (0.20 mmol). The reaction mixture is irradiated with microwave radiation at 120 °C for 20 min. It is filtered and then extracted with ethylacetate. The crude products are purified by column chromatography. The final products, **13ap-13as** and **13bp-13bs** are obtained as white solids.

(*S*)-(+)-*2-methyl-3-(octyloxy)-3-oxopropyl-4'-(3-(4-(benzyloxycarbonyl)phenyl)-1,2,4-oxadiazol-5-yl)biphenyl-4-carboxylate***(13ap,** Ph.Ox.C*C$_8$**):** Yield 34.0%; ^{1}H NMR (400 MHz, $CDCl_3$) δ (ppm) 0.85 (t, *J* = 7.00 Hz, 3H),1.22 - 1.30 (m, 10H), 1.32 (d, *J* = 7.08, 3H),

1.59 - 1.64 (m, 2H), 2.96 - 3.02 (m, 1H), 4.14 (t, *J* = 6.52 Hz, 2H), 4.46 - 4.51 (m, 2H), 5.41 (s, 2H), 7.37 - 7.44 (m, 3H), 7.49 (d, *J* = 7.20 Hz, 2H), 7.73 (d, *J* = 8.32 Hz, 2H), 7.82 (d, *J* = 8.32 Hz, 2H), 8.13 (d, *J* = 8.28 Hz, 2H), 8.23 (d, *J* = 8.48 Hz, 2H), 8.28 (d, *J* = 8.32 Hz, 2H), 8.32(d, *J* = 8.36 Hz, 2H); IR (cm^{-1}): 3066 (Ar C - H), 2924, 2853 (C - H), 1712 (C = O), 1611, 1460 (C - C in ring), 1356 (C - H bend), 1273 (C - O - C), 1179, 1106 (C - O), 749 (C - H rock); Elemental Analysis: calculated for $C_{41}H_{42}N_2O_7$ (674.78), C 72.98, H 6.27, N 4.15; Found: C 73.08, H 6.21, N 4.10%.

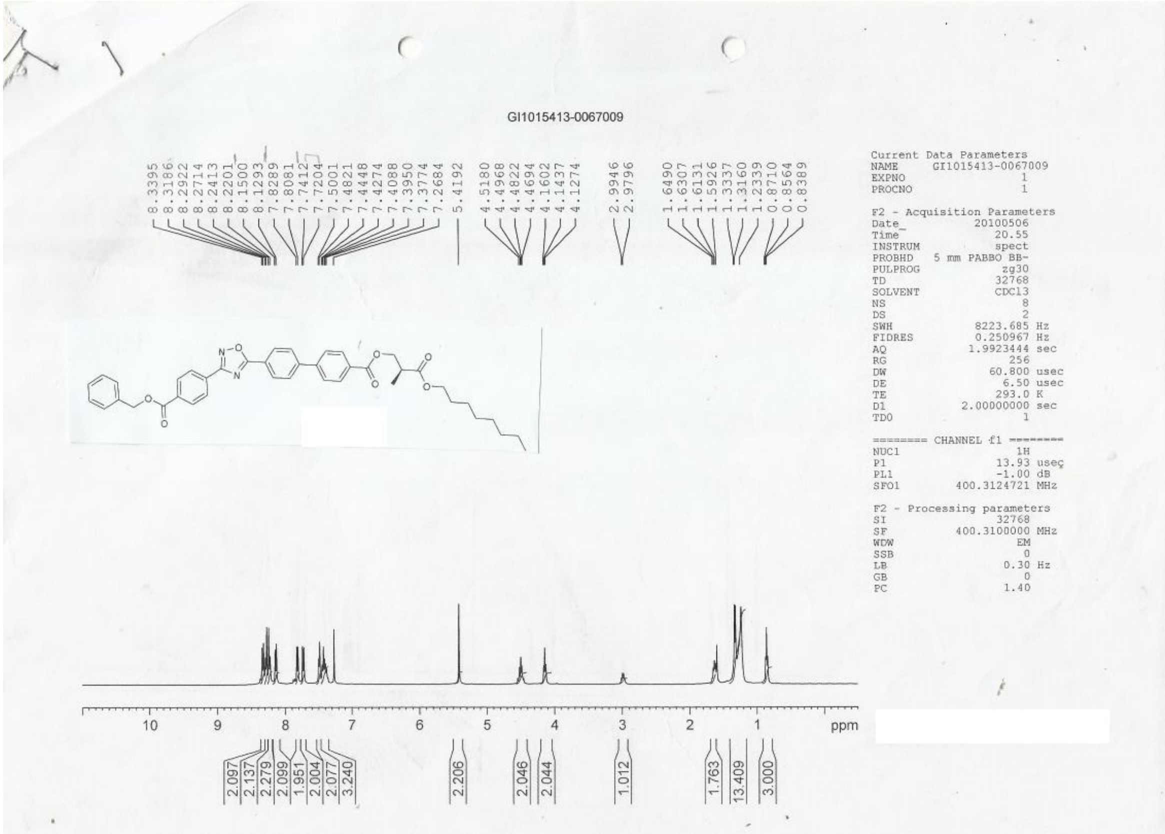

Fig. 6: ^{1}H NMR of **13ap**

(*S*)-(+)-*2-methyl-3-(nonyloxy)-3-oxopropyl-4'-(3-(4-(benzyloxycarbonyl)phenyl)-1,2,4-oxadiazol-5-yl)biphenyl-4-carboxylate* **(13aq,** Ph.Ox.C*C_9**):** Yield 36.2%; ^{1}H NMR (400 MHz, $CDCl_3$) δ (ppm) 0.87 (t, *J* = 7.14 Hz, 3H),1.22 - 1.27 (m, 12H), 1.32 (d, *J* = 7.12, 3H), 1.59 - 1.64 (m, 2H), 2.98 - 3.02 (m, 1H), 4.11 (t, *J* = 6.68 Hz, 2H), 4.49 - 4.53 (m, 2H), 5.41 (s, 2H), 7.37 - 7.46 (m, 3H), 7.49 (d, *J* = 7.54 Hz, 2H), 7.72 (d, *J* = 6.76 Hz, 2H), 7.82 (d, *J*= 7.46 Hz, 2H), 8.13 (d, *J* = 8.40 Hz, 2H), 8.22 (d, *J* = 8.56 Hz, 2H), 8.29 (d, *J* = 8.64 Hz, 2H), 8.33(d, *J* = 8.26 Hz, 2H); IR (cm^{-1}): 3064 (Ar C - H), 2927, 2854 (C - H), 1718 (C = O), 1614, 1462 (C - C in ring), 1357 (C - H bend), 1274 (C - O - C), 1176, 1108 (C - O), 755 (C

- H rock); Elemental Analysis: calculated for $C_{42}H_{44}N_2O_7$ (688.80), C 73.24, H 6.44, N 4.07; Found: C 73.14, H 6.40, N 3.92%.

(*S*)-(+)-*2-methyl-3-(decyloxy)-3-oxopropyl-4'-(3-(4-(benzyloxycarbonyl)phenyl)-1,2,4-oxadiazol-5-yl)biphenyl-4-carboxylate* **(13ar,** Ph.Ox.C*C_{10}**):** Yield 31.3%; ^{1}H NMR (400 MHz, $CDCl_3$) δ (ppm) 0.86 (t, *J* = 7.04 Hz, 3H), 1.22 - 1.29 (m, 14H), 1.32 (d, *J* = 7.12, 3H), 1.58 - 1.64 (m, 2H), 2.96 - 3.01 (m, 1H), 4.14 (t, *J* = 6.56 Hz, 2H), 4.48 - 4.52 (m, 2H), 5.41 (s, 2H), 7.37 - 7.44 (m, 3H), 7.49 (d, *J* = 6.76 Hz, 2H), 7.73 (d, *J* = 8.36 Hz, 2H), 7.81 (d, *J* = 8.44 Hz, 2H), 8.13 (d, *J* = 8.40 Hz, 2H), 8.23 (d, *J* = 8.48 Hz, 2H), 8.28 (d, *J* = 8.52 Hz, 2H), 8.33 (d, *J* = 8.40 Hz, 2H); IR (cm^{-1}): 3065 (Ar C - H), 2919, 2851 (C - H), 1721 (C = O), 1613, 1493 (C - C in ring), 1415 (C - H bend), 1274 (C - O - C), 1139, 1115 (C - O), 754 (C - H rock); Elemental Analysis: calculated for $C_{43}H_{46}N_2O_7$ (702.83), C 73.48, H 6.60, N 3.99; Found: C 73.41, H 6.38, N 3.92%.

(*S*)-(+)-*2-methyl-3-(dodecyloxy)-3-oxopropyl-4'-(3-(4-(benzyloxycarbonyl)phenyl)-1,2,4-oxadiazol-5-yl)biphenyl-4-carboxylate* (**13as,** Ph.Ox.C*C_{12}): Yield 34.7%; ^{1}H NMR (400 MHz, $CDCl_3$) δ (ppm) 0.86 (t, *J* = 7.08 Hz, 3H), 1.22 - 1.28 (m, 18H), 1.32 (d, *J* = 7.12, 3H), 1.59 - 1.63 (m, 2H), 2.97 - 3.03 (m, 1H), 4.12 (t, *J* = 6.64 Hz, 2H), 4.48 - 4.51 (m, 2H), 5.41 (s, 2H), 7.37 - 7.47 (m, 3H), 7.49 (d, *J* = 7.54 Hz, 2H), 7.72 (d, *J* = 6.76 Hz, 2H), 7.81 (d, *J* = 7.48 Hz, 2H), 8.14 (d, *J* = 8.44 Hz, 2H), 8.23 (d, *J* = 8.56 Hz, 2H), 8.28 (d, *J* = 8.64 Hz, 2H), 8.32 (d, *J* = 6.76 Hz, 2H); IR (cm^{-1}): 3063 (Ar C - H), 2918, 2850 (C - H), 1714 (C = O), 1611, 1459 (C - C in ring), 1416 (C - H bend), 1269 (C - O - C), 1203, 1102 (C - O), 745 (C - H rock); Elemental Analysis: calculated for $C_{45}H_{50}N_2O_7$ (730.88), C 73.95, H 6.90, N 3.83; Found: C 73.90, H 6.83, N 3.74%.

(*S*)-(+)-*2-methyl-3-(octyloxy)-3-oxopropyl-4'-(3-(4-(dodecyloxycarbonyl)-phenyl)-1,2,4-oxadiazol-5-yl)biphenyl-4-carboxylate* **(13bp,** C_{12}.Ox.C*C_8**):** Yield 24.0%; ^{1}H NMR (400 MHz, $CDCl_3$) δ (ppm) 0.87 (t, *J* = 7.04 Hz, 6H), 1.23 - 1.27 (m, 26H), 1.33 (d, *J* = 7.16 Hz, 3H), 1.43 - 1.47 (m, 2H), 1.56 - 1.64 (m, 2H), 1.79 - 1.84 (m, 2H), 2.96 - 3.01 (m, 1H), 4.14 (t, *J* = 6.0 Hz, 2H), 4.36 (t, *J* = 6.68 Hz, 2H), 4.44 - 4.54 (m, 2H), 7.73 (dd, *J* = 1.76, 6.72 Hz, 2H), 7.81 (dd, *J* = 1.76, 6.72 Hz, 2H), 8.13 (dd, *J* = 1.68,6.72 Hz, 2H), 8.18 (dd, *J* = 1.84, 6.8 Hz, 2H), 8.27 (dd, *J* = 1.72, 6.68 Hz, 2H), 8.32 (dd, *J* = 1.76, 6.76 Hz, 2H); IR (cm^{-1}): 3076 (Ar C - H), 2918, 2851 (C - H), 1719 (C = O), 1613, 1471 (C - C in ring), 1416 (C - H bend), 1278 (C - O - C), 1207, 1121 (C - O), 747 (C - H rock); Elemental Analysis: calculated for $C_{46}H_{60}N_2O_7$ (752.97), C 73.37, H 8.03, N 3.72; Found: C 73.25, H 8.11, N 3.62%.

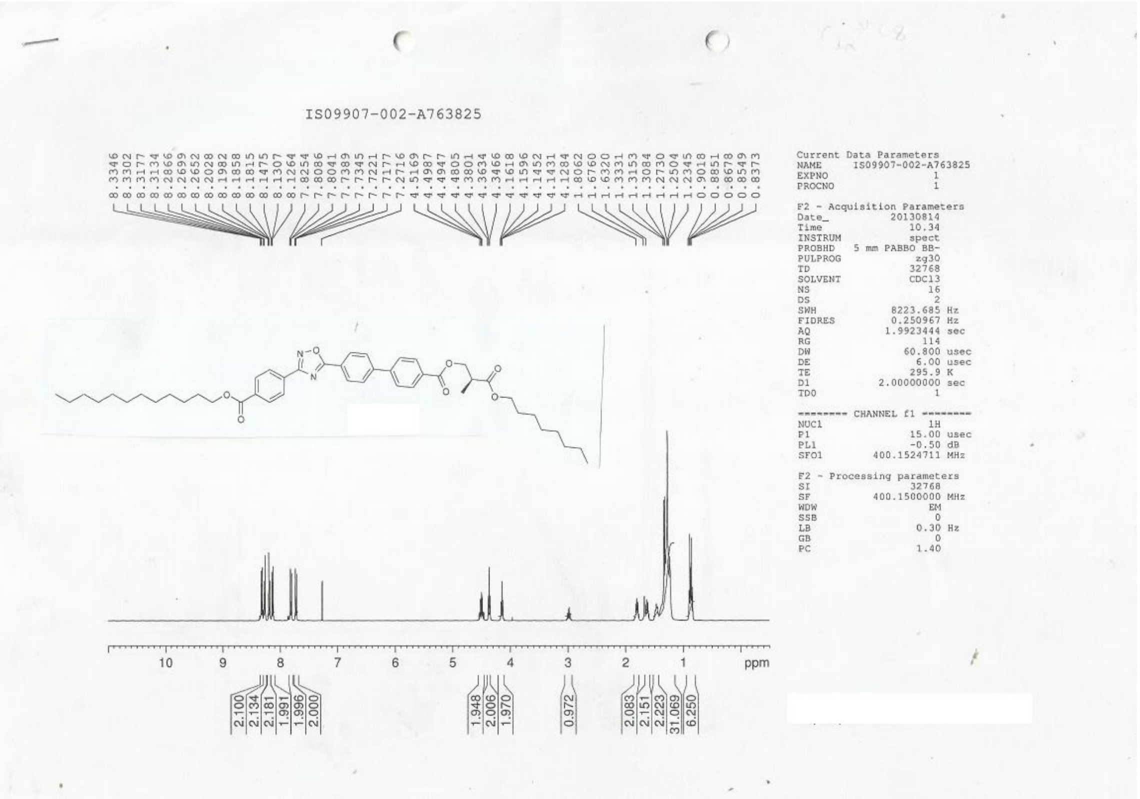

Fig. 7: ^{1}H NMR of **13bp**

(S)-(+)-2-methyl-3-(nonyloxy)-3-oxopropyl-4'-(3-(4-(dodecyloxycarbonyl)phenyl)-1,2,4-oxadiazol-5-yl)biphenyl-4-carboxylate (**13bq,** C_{12}.Ox.C*C_9**):** Yield 25.9%; ^{1}H NMR (400 MHz, $CDCl_3$) δ (ppm) 0.88 (t, *J* = 6.94 Hz, 6H), 1.23 - 1.28 (m, 28H), 1.32 (d, *J* = 7.18Hz, 3H), 1.34 - 1.38 (m, 2H), 1.57 - 1.65 (m, 2H), 1.80 - 1.85 (m, 2H), 2.98 - 3.04 (m, 1H), 4.15 (t, *J* = 6.20 Hz, 2H), 4.37 (t, *J* = 6.74 Hz, 2H), 4.47 - 4.56 (m, 2H), 7.72 (dd, *J* = 1.72, 6.76 Hz, 2H), 7.81 (dd, *J* = 1.76, 6.72 Hz, 2H), 8.13 (dd, *J* = 1.68, 6.72 Hz, 2H), 8.19 (dd, *J* = 1.84, 6.8 Hz, 2H), 8.26 (dd, *J* = 1.72, 6.68 Hz, 2H), 8.32 (dd, *J* = 1.76, 6.76 Hz, 2H); IR (cm^{-1}): 3074 (Ar C - H), 2920, 2853 (C - H), 1711 (C = O), 1612, 1468 (C - C in ring), 1414 (C - H bend), 1277 (C - O - C), 1208, 1126 (C - O), 744 (C - H rock); Elemental Analysis: calculated for $C_{47}H_{62}N_2O_7$ (767.00), C 73.60, H 8.15, N 3.65; Found: C 73.76, H 8.09, N 3.71%.

(S)-(+)-2-methyl-3-(decyloxy)-3-oxopropyl-4'-(3-(4-(dodecyloxycarbonyl)phenyl)-1,2,4-oxadiazol-5-yl)biphenyl-4-carboxylate (**13br,** C_{12}.Ox.C*C_{10}**):** Yield 29.4%; ^{1}H NMR (400 MHz, $CDCl_3$) δ (ppm) 0.87 (t, *J* = 7.08 Hz, 6H), 1.22- 1.29 (m, 30H), 1.32(d, *J* = 7.12Hz, 3H), 1.45- 1.49(m, 2H), 1.61- 1.65(m, 2H), 1.79- 1.83(m, 2H), 2.96- 3.11(m, 1H), 4.14 (t, *J* = 6.60 Hz, 2H), 4.37 (t, *J* = 6.68 Hz, 2H), 4.38- 4.52 (m, 2H), 7.73 (dd, *J* = 1.64, 8.44 Hz, 2H), 7.82 (dd, *J* = 1.68, 8.48 Hz, 2H), 8.14 (dd, *J* = 1.72, 8.44 Hz, 2H), 8.20 (dd, *J* = 1.80, 8.56

Hz, 2H), 8.28 (dd, J = 1.88, 7.84 Hz, 2H), 8.33 (d, J = 8.48 Hz, 2H); IR (cm^{-1}): 3075 (Ar C - H), 2919, 2851 (C - H), 1722 (C = O), 1612, 1468 (C - C in ring), 1415 (C - H bend), 1278 (C - O - C), 1209, 1127 (C - O), 745 (C - H rock); Elemental Analysis: calculated for $C_{48}H_{64}N_2O_7$ (781.03), C 73.81, H 8.26, N 3.59; Found C 73.68, H 8.19, N 3.45%

(*S*)-(+)-*2-methyl-3-(dodecyloxy)-3-oxopropyl-4'-(3-(4-(dodecyloxycarbonyl)phenyl)-1,2,4-oxadiazol-5-yl)biphenyl-4-carboxylate* **(13bs,** C_{12}.Ox.C*C_{12}**)**: Yield 28.2%; ^{1}H NMR (400 MHz, $CDCl_3$) δ (ppm) 0.88 (t, J = 7.06 Hz, 6H), 1.25 - 1.29 (m, 34H), 1.32 (d, J = 7.12Hz, 3H), 1.45 - 1.38 (m, 2H), 1.61 - 1.64 (m, 2H), 1.79 - 1.82 (m, 2H), 2.96 - 3.01 (m, 1H), 4.14 (t, J = 5.88 Hz, 2H), 4.36 (t, J = 6.68 Hz, 2H), 4.45 - 4.54 (m, 2H), 7.73 (d, J = 8.36 Hz, 2H), 7.82 (d, J = 8.36 Hz, 2H), 8.14 (d, J = 8.32 Hz, 2H), 8.20 (d, J = 8.44 Hz, 2H), 8.28 (d, J = 8.28 Hz, 2H), 8.33 (d, J = 8.36 Hz, 2H); IR (cm^{-1}): 3072 (Ar C - H), 2915, 2848 (C - H), 1714 (C = O), 1613, 1469 (C - C in ring), 1415 (C - H bend), 1277 (C - O - C), 1207, 1115 (C - O), 745 (C - H rock); Elemental Analysis: calculated for $C_{50}H_{68}N_2O_7$ (809.08), C 74.22, H 8.47, N 3.46; Found: C 74.12, H 8.37, N 3.52%.

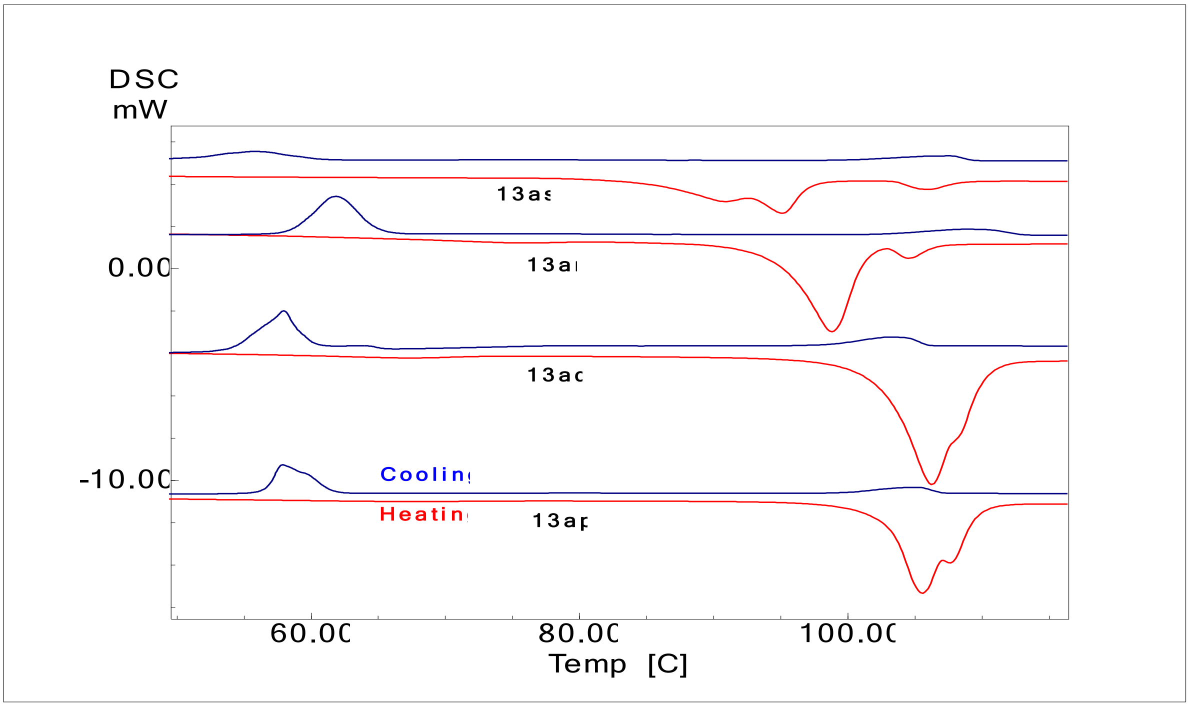

Fig. 8: DSC thermograms (1st run @ 10 °C/min.) of **13a** series

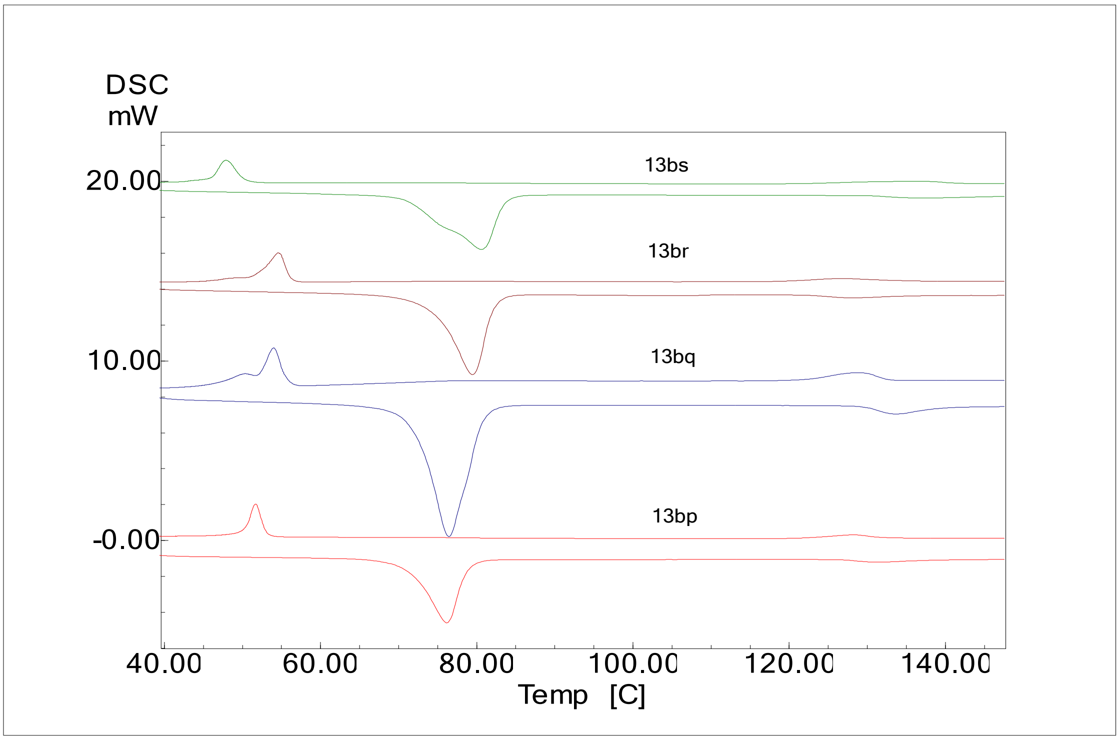

Fig. 9: DSC thermograms (1st run @ 10 °C/min.) of **13b** series

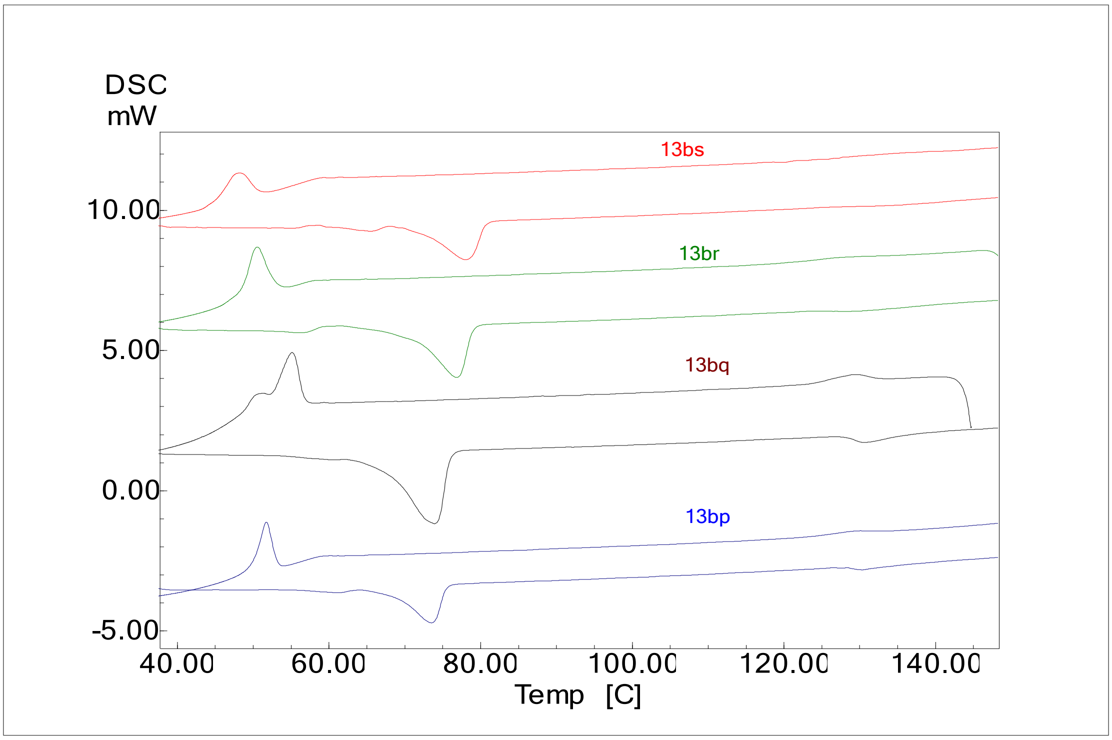

Fig. 10: DSC thermograms (2nd run @ 5 °C/min.) of **13b** series

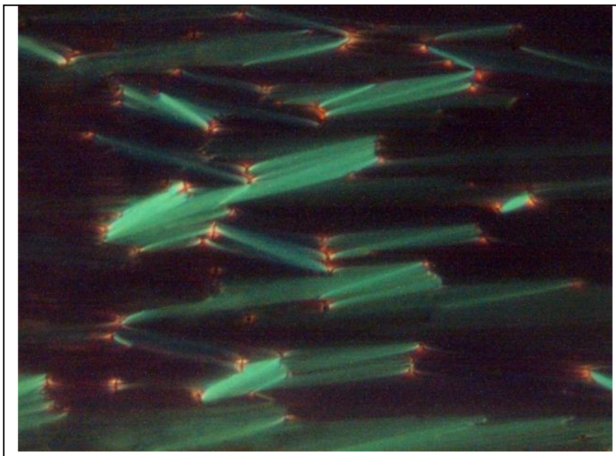

at 127.6 °C

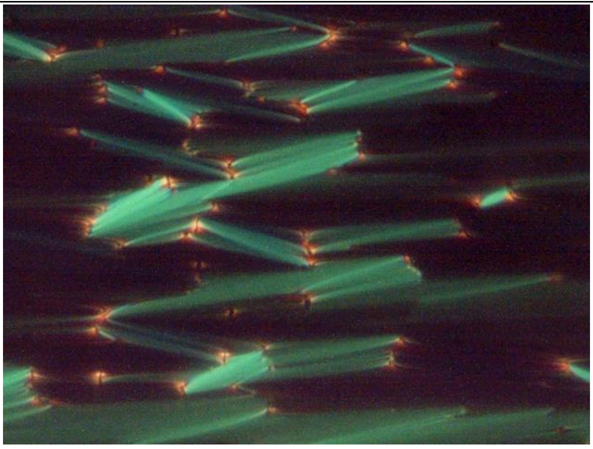

at 112.4 °C

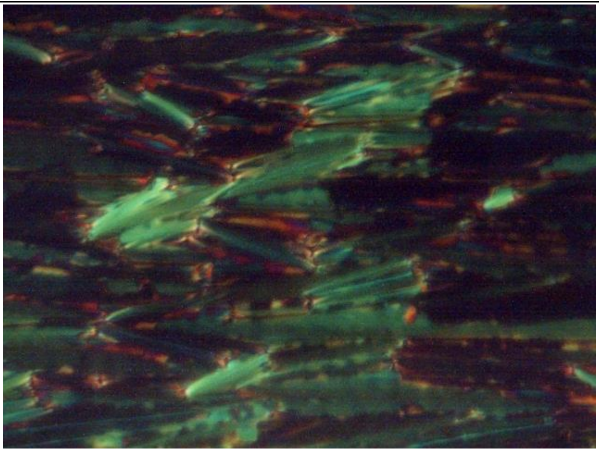

at 83.2 °C

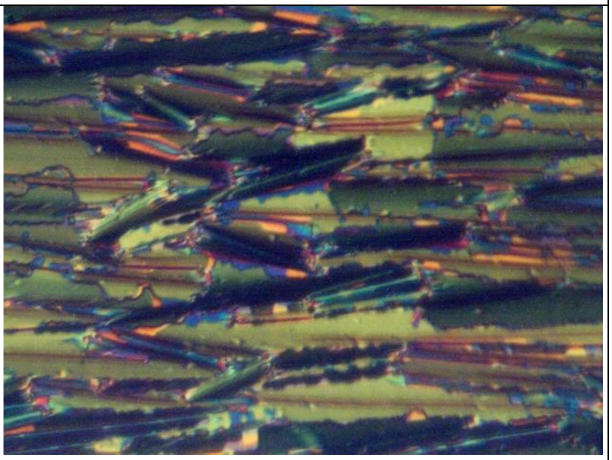

at 79.4 °C

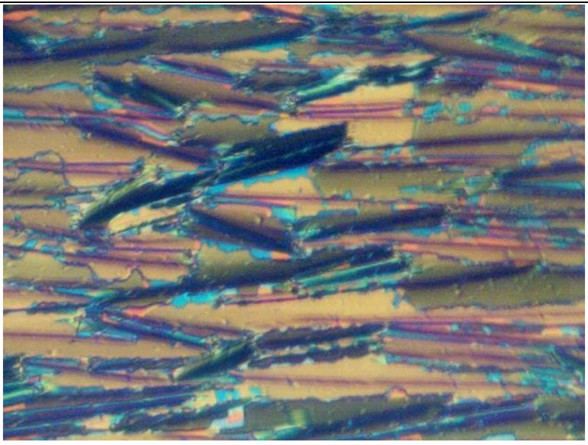

at 67.6 °C

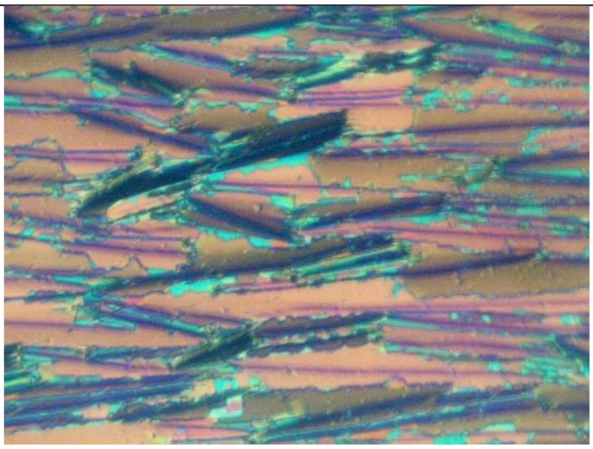

at 60.8 °C

Fig. 11: POM textures exhibited by **13bp** at different temperatures

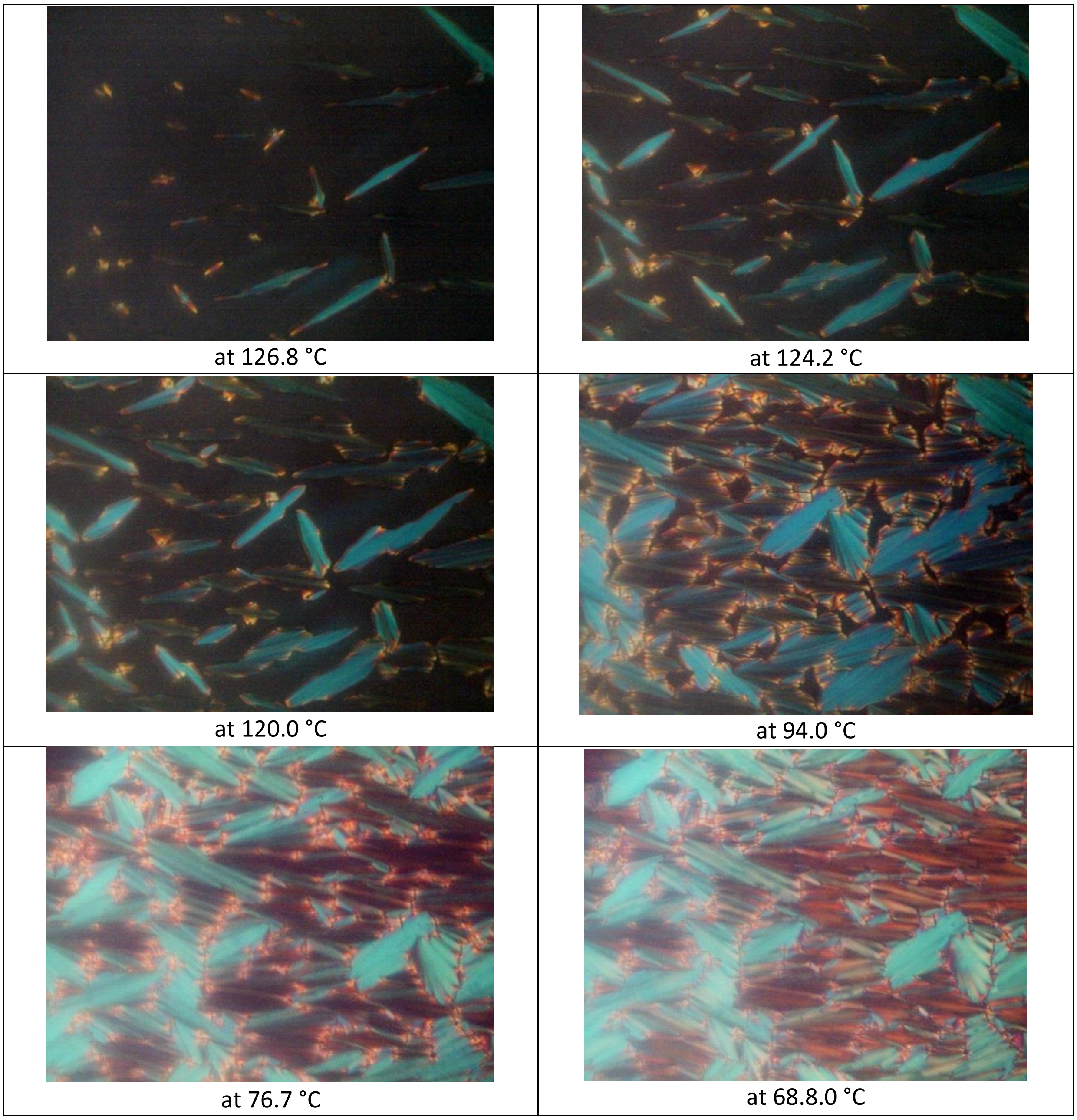

Fig. 12: POM textures exhibited by **13br** at different temperatures

*****
